# Virulence traits and bacterial interactions within the complex microbial population in urinary double-J catheters

**DOI:** 10.3389/fmicb.2025.1624743

**Published:** 2025-07-10

**Authors:** Juan Vicente Farizano, Emilia Castagnaro, Julián Thomas Arroyo-Egea, Juan Daniel Aparicio, Alicia Cecilia Vallejos, Elvira María Hebert, Lucila Saavedra, Viviana Andrea Rapisarda, Josefina María Villegas, Mariana Grillo-Puertas

**Affiliations:** ^1^Instituto Superior de Investigaciones Biológicas (INSIBIO), CONICET-UNT, and Instituto de Química Biológica “Dr. Bernabé Bloj”, Facultad de Bioquímica, Química y Farmacia, UNT, San Miguel de Tucumán, Argentina; ^2^Planta Piloto de Procesos Industriales Microbiológicos (PROIMI-CONICET), Tucumán, Argentina; ^3^Hospital Eva Perón, Ministerio de Salud Pública de Tucumán, Tucumán, Argentina; ^4^Centro de Referencia para Lactobacilos (CERELA-CONICET), Tucumán, Argentina

**Keywords:** uropathogens, catheters, biofilm, virulence, bacterial interaction, antibiotic resistance

## Abstract

Indwelling devices, such as double-J stents, are commonly used in urological surgery and are often associated with a high risk of urinary tract infections (UTIs) due to biofilm-related complications. In this study, we characterized 27 clinical bacterial isolates from double-J catheters, including *Staphylococcus* spp., *Enterococcus faecalis, Klebsiella pneumoniae, Escherichia coli*, and *Bacillus* spp., to investigate their pathogenic potential. Our findings revealed that strong biofilm producers (*E. coli, S. aureus*, and *B. subtilis*) exhibited robust extracellular matrix synthesis. Notably, multidrug resistance was observed in *E. coli, K. pneumoniae*, and *E. faecalis*. Mixed-culture experiments demonstrated that *Bacillus* spp. enhanced the biofilm formation of uropathogens, suggesting a potential impact on clinical outcomes. The characterization of the polymicrobial population colonizing double-J catheters, as conducted in this study, is essential for understanding the complexity and clinical behavior of biofilm-related infections associated with medical devices. Additionally, our results highlight the clinical relevance of underreported genera, such as *Bacillus*, which are often overlooked in routine clinical diagnostics. Gaining insights into the interaction mechanisms and survival strategies of several bacterial species colonizing double-J catheters may help shift current paradigms in understanding catheter-related infections.

## Introduction

Urinary tract infections (UTIs) affect a large number of people, with prevalence varying by age group, sex, and the presence of risk factors (Echverría-Zarate et al., 2006; Pigrau, 2013; Delcaru et al., 2016; Klein and Hultgren, 2020). The high incidence of UTIs, along with increased recurrence rates and the potential for complications, contributes to the cost of medical treatment, amounting to billions of dollars in healthcare expenditures (Cepas et al., 2019). The primary cause of UTIs is the invasion of the urinary tract by microorganisms known as uropathogens (UP). The leading etiologic agent in human UTIs is uropathogenic *E. coli* (UPEC), responsible for up to 80% of all cases (Ronald, 2002). Other microorganisms, such as *Staphylococcus* spp., *Proteus mirabilis, Proteus vulgaris, Klebsiella* spp., *Enterococcus faecalis*, and *Pseudomonas aeruginosa*, account for the remaining 20% of infections (Nicolle, 2000; Flores-Mireles et al., 2015).

The risk of developing an UTI significantly increases with the use of indwelling devices, as they disrupt host defense mechanisms and facilitate the access of UP to the bladder or the upper urinary tract (Wu et al., 2015). The double-J stent is a commonly used device in urological surgery, serving dual functions by providing both support and drainage (Ozturk, 2015; Gauhar et al., 2022; Lin et al., 2022). However, ureteral stents are often associated with severe complications, primarily due to biofilm formation on the stent surface, which can lead to infections. These infections may result in bacteremia, renal function deterioration, pyelonephritis, or even mortality due to sepsis (Ranganathan et al., 2009). Biofilms developing on ureteral stents can originate from the urinary tract microbiota or from contamination during stent insertion (Bossa et al., 2017). The internal environment of ureteral stents provides an ideal medium for bacterial adhesion, colonization, and biofilm formation (Zumstein et al., 2017). An additional challenge with these stents lies in diagnosis, as colonizing bacteria may not be detected in urine or prostatic secretion samples (Klis et al., 2009). Therefore, identifying the pathogenic bacteria involved in double-J catheter colonization is crucial for effective treatment of these infections.

Bacterial populations have the capacity to rapidly adapt to changes in their environmental surroundings (O'Toole and Kolter, 1998; Monds and O'Toole, 2009). The ability of UP strains to cause various types and severities of diseases depends on the expression of multiple virulence traits, including biofilm formation, motility, hemolytic activity, expression of several virulence genes related to adhesin production, toxins, siderophores, and secretion systems, among others (Blanco et al., 2002; Johnson, 2002). UP are often found in a sessile state on the intra- and/or extraluminal surfaces of urinary catheters removed from patients with UTIs (Li et al., 2023).

Over the years, several reports have shown that the majority of biofilms formed on long-term catheters are polymicrobial, with an average of 2–5 microbial species isolated per catheter, predominantly comprising Gram-negative bacteria (Nicolle, 2012; Azevedo et al., 2017; Chua et al., 2017; Flores-Mireles et al., 2019; Zboromyrska et al., 2019). UP isolates from polymicrobial biofilms in catheterized patients have exhibited significant resistance to commonly used antibiotics, thereby increasing the risk of developing severe infections (Croxall et al., 2011; Kazi et al., 2015). In the context of the urinary tract, the presence of multiple microorganisms in a midstream urine sample is often attributed to contamination from periurethral or vaginal microbiota (Hooton, 2012; Kline and Lewis, 2016). Polymicrobial samples may also be classified as negative cultures if they lack a single dominant species, or they may be categorized as containing secondary pathogens or doubtful pathogens, depending on the level of colonization. As a result, the virulence and complexity of the often underestimated polymicrobial colonization of double-J catheters remain poorly characterized. In this context, this study aimed to investigate the virulence-associated phenotypic traits and antimicrobial resistance of bacteria isolated from double-J catheters, as well as to explore the interactions within mixed biofilms formed by co-isolated bacterial pairs. Through the comprehensive characterization of 27 isolates from this complex niche, we analyzed the virulent potential and interactions between known UP and understudied species such as *Bacillus* spp. Such knowledge is crucial for better managing catheter-associated infections, which significantly impact patient outcomes and healthcare systems.

## Materials and methods

### Catheter processing and UP isolation

Samples were taken from eight double-J-type urinary catheters provided by a specialized private urological center in San Miguel de Tucumán, Argentina. In all cases, 30–60 days urinary catheters were collected from adult male and female patients without UTI symptoms and with a negative urine culture. Each catheter was processed under sterile conditions to remove adherent and non-adherent bacteria, as reported by Mandakhalikar et al. (2018). Briefly, to extract unattached cells, four washes with physiological saline (PS) solution were performed. To extract adhered bacteria from catheters, the vortexing–sonication–vortexing (V-S-V) method was carried out (Mandakhalikar et al., 2018). Both unattached and attached bacteria were concentrated by centrifugation for 10 min at 7012 × *g*. Then, cells were resuspended in PS and seeded with a Digralsky spatula onto Cystine Lactose Electrolyte Deficient Agar (CLED-agar, Britania) plates. The plates were incubated for 24–48 h at 37°C to evaluate microbial growth. Colonies with different morphologies were purified using the quadrant streak method on CLED-agar medium. Pure isolates of potential UP were preserved for subsequent identification.

### Identification of UP isolates

To identify the obtained UP isolates, genomic DNA was extracted using the organic solvent extraction method reported by Sambrook et al. (1989). The extracted genomic DNA was quantified using a NanoDrop spectrophotometer (UV/Vis nano spectrophotometer, Nabi).

Then, amplification of the 16S rRNA gene was conducted and sequenced to identify the genus of the obtained UP isolates (Sanger sequencing, Macrogen Inc.). Moreover, the identification of UP at the species level was carried out according to the standard methods and recommendations of the International Committee of Clinical Laboratories (CLSI, 2020). Standard biochemical tests and confirmation by MALDI-TOF mass spectrometry techniques were also used for identification (Bruker Daltonics; Perilla et al., 2009; Murray et al., 2021).

### Culture conditions

Isolates were routinely grown under aerobic conditions in BHI medium at 37°C with shaking (180 rpm) or in static growth at 37°C on CLED-agar plates. Bacterial growth was monitored by measuring the absorbance at 600 nm (A_600 nm_) to determine turbidity in a liquid medium or by observing the appearance of isolated colonies in a solid medium. MacConkey (Britania), Luria Broth (LB, Sigma-Aldrich), M63 (in g/L: 2.0 ammonium sulfate, 13.6 monopotassium phosphate, 0.005 ferrous sulfate heptahydrate, 0.12 magnesium sulfate, 2.0 glycerol), Tryptic Soy Broth (TSB, Britania), Brain Heart Infusion (BHI, Britania) and urine media were used, depending on the assay. Human urine media was prepared according to Eberly et al. (2020). Briefly, urine was collected in equal volumes from male and female healthy volunteers and filtered through a 0.22 μm filter prior to use. Healthy volunteers were classified as individuals who are urologically asymptomatic, not menstruating, and those who have not taken antibiotics in the last 90 days.

### Biofilm formation and quantification

Biofilm formation was assessed by measuring the ability of cells to adhere and grow in 96-well polystyrene plates using various culture media (O'Toole and Kolter, 1998). Briefly, 24-h cultures of all isolates were washed in PS, adjusted to an A_600 nm_ of 1.0, and then diluted to an A_600 nm_ of 0.1 in the corresponding media. Suspensions were loaded into multiwell plates and incubated under static conditions at 30°C for the specified times in each assay. Then, unattached (planktonic) cells were removed, and wells were washed three times with distilled water. The quantification of adherent cells or biofilm biomass was performed as described by O'Toole and Kolter (1998). Each condition was performed in quadruplicate, and each experiment was repeated at least five times.

UP isolates were classified according to their biofilm formation capacity after 72 h, following the Stepanovic criteria (Stepanovic et al., 2007). The A_595nm_ cut-off value (A_595nm_C) was defined as the average value of the blank absorbance plus three standard deviations. The blank was the non-inoculated medium. Consequently, the following classification criteria were established: A_595nm_ ≤ A_595nm_C, non-biofilm producers (–); A_595nm_C < A_595nm_ ≤ 2 fold A_595nm_C, weak biofilm producers (+); 2 fold A_595nm_C < A_595nm_ ≤ 4 fold A_595nm_C, moderate biofilm producers (++); 4 fold A_595nm_C < A_595nm_ ≤ 8 fold A_595nm_C, strong biofilm producers (+++); and A_595nm_ > 8 fold A_595nm_C, robust biofilm producers (++++).

### Microscopy biofilm visualization

Selected isolates were grown under biofilm-forming conditions in 6-well polystyrene plates containing M63 medium. Each well was previously fitted with a sterile glass coverslip (provided by the microscopy facilities). After 72 h at 30°C, non-adherent cells were gently removed, and the coverslips were washed with distilled water and air-dried for 10 min. For confocal laser scanning microscopy (CLSM), biofilms were stained with 20 μM DAPI (Sigma) in 0.1 M Tris-HCl buffer (pH 8) for 10 min (in dark conditions), followed by distilled water washes. Samples were fixed with 4% paraformaldehyde (for 20 min), rinsed with PBS, and imaged using a Zeiss LSM800 microscope. For scanning electron microscopy (SEM), biofilms on coverslips were fixed with a 2.5% glutaraldehyde and 2.5% paraformaldehyde (v/v) solution, dehydrated through an acetone/ethanol series, and sputter-coated with gold using a JEOL JFC-1100 ion coater. Samples were then mounted on aluminum stubs and imaged with a Carl Zeiss SUPRA-55 SEM at resolutions of 1.0 nm (15 kV) and 1.7 nm (1 kV) in high-vacuum mode or 2 nm (30 kV) in variable-pressure mode.

### Colony morphotypes: amyloid-like fiber and cellulose production

Colony morphology and dye-binding may serve as indicators of several physiological and metabolic states in microbes (Martin-Rodriguez et al., 2021). To analyze colony morphology, culture plates supplemented with Congo Red (CR, Cicarelli) and Brilliant Blue (BB, Sigma-Aldrich) were used, as described by Da Re and Ghigo (2006). Briefly, BHI cultures of all isolates were grown overnight (ON) at 37°C, washed, and diluted to an A_600 nm_ of 0.1 in PS. Subsequently, 5 μL of these suspensions were spotted on LB-agar plates (low salt) supplemented with 40 μg/mL CR and 20 μg/mL BB. Plates were incubated at 30°C for 96 h and monitored every 24 h. Colony phenotype analysis was performed for each bacterial genus, as per previous reports. For *E. coli* and *K. pneumoniae*, the analysis was according to Bokranz et al. (2005): *ras* (red and smooth colonies) and *pas* (pink and smooth colonies) morphotypes. For *Staphylococcus* spp. isolates, the results were interpreted as reported by Arciola et al. (2002): reddish-black colonies with a rough and dry consistency were considered to be biofilm-related extracellular matrix-producing strains. The morphology of *E. faecalis* strains was analyzed as reported by Torres-Rodríguez et al. (2020), where strains with black and rough colonies are considered biofilm producers, and strains with red or white colonies are considered non-producers. For *Bacillus* spp., bacteria that bind CR dye have previously been classified as producers of functional bacterial amyloid fibers (Romero et al., 2010). The presence of amyloid-like fiber was denoted as “+.”

Cellulose production was assessed as described by White et al. (2006). For this, BHI cultures of each isolate were grown ON at 37°C, washed, and diluted to an A_600 nm_ of 0.1 in PS. Subsequently, 5 μL of the isolates were spotted on LB-agar plates supplemented with 50 μg/mL Calcofluor White (CW, Sigma-Aldrich) dye. The plates were incubated at 30°C for 96 h and monitored every 24 h. Cellulose production was qualitatively assessed by observing the fluorescence of colonies when irradiated with UV light. Fluorescent colonies were denoted as +.

### Motility assay

Bacterial motility was assessed according to Ulett et al. (2006), with minor modifications. Briefly, ON BHI cultures were washed and diluted to an A_600 nm_ of 0.1 in PS. Suspensions were then seeded onto semi-solid LB-agar plates (0.3%) using a sterile toothpick. The plates were incubated at 30°C, and the colony diameter was evaluated over time. Isolates with colony diameters between 0.5 and 1 cm were denoted as “+” and those with diameters >1 cm as “++.”

### Hemolytic capacity

The isolates' hemolytic activity was assessed on blood agar plates using the method described by Gerhardt et al. (1994). Briefly, isolates were grown ON in BHI medium, and then the cells were washed and diluted to an A_600 nm_ of 0.1 in PS. Aliquots of the bacterial suspensions were plated on blood agar medium and incubated for 24 h at 30°C. A β-hemolytic reaction involves the complete lysis of red blood cells, resulting in a clear area on the agar surrounding the colony, known as total hemolysis. In contrast, an α-hemolytic reaction occurs when the hemoglobin in red blood cells is converted to methemoglobin, resulting in a greenish tint in the agar surrounding the colonies. Finally, the absence of hemolysis or discoloration is referred to as γ-hemolysis (Buxton, 2005).

### Siderophore production assay

The siderophore production was determined using a qualitative technique adapted in our laboratory, based on the color change of Cromoazurol S (CAS, Sigma-Aldrich). Strains were cultured ON in M9 minimal medium (Sigma-Aldrich) supplemented with 0.2% glucose, with shaking at 37°C. Subsequently, 5 μL aliquots of each cell culture were seeded onto M9 minimal medium supplemented with 0.2% glucose and incubated at 37°C for 48 h. Then, an overlay of semisolid CAS medium (Cromoazurol S, 60.5 mg; piperazine acid, 72.9 mg; FeCl_3_, 1 mM; dissolved in HCl at 10 mM; 10 mL of this solution per 1 L) was poured onto the grown plates. Isolates capable of producing siderophores exhibited a color change in the CAS medium from blue to yellowish around the colony.

### Hydrogen peroxide, acid, and human serum tolerance assays

Stressor susceptibility was carried out according to Shea et al. (2022). Briefly, bacterial cultures were incubated ON in BHI medium at 37°C. Cultures were normalized to 0.5 McFarland (~1.5 × 10^8^ CFU mL^−1^) in 1 mL of either BHI, BHI containing fresh 0.2% H_2_O_2_, or BHI buffered to pH 7, 5, or 2.5. Samples were immediately vortexed and incubated either for 15 or 60 min at room temperature for the H_2_O_2_ tolerance assay or for 1 h at 37°C with aeration for the acid tolerance assay. At each time point, suspensions were serially diluted in PS to further determine CFU mL^−1^ in CLED-agar medium. For human serum tolerance, ON BHI cultures (1 mL) were pelleted by centrifugation, and 0.5 McFarland suspensions were prepared in sterile PS. The suspensions were diluted 1:200 in either 100% human serum or 100% heat-inactivated human serum (Sigma-Aldrich). The mixture was incubated for 1 h at 37°C, and then the number of CFU mL^−1^ was calculated by serially diluting the bacterial-serum suspension and plating it on CLED-agar medium. The CFU mL^−1^ of the bacterial inoculum was calculated by serially diluting the bacterial suspension and plating it on CLED-agar medium.

### Antibiotic susceptibility

Antibiograms were performed for each UP using the disc diffusion method on Müller-Hinton agar medium (MH-agar, Britania; CLSI, 2020). The choice of antimicrobial agents is based on national and international recommendations for urinary infections in adults, covering both inpatient and outpatient settings (WHO, 2022). The antibiotics (ATB) used for Gram-negative and Gram-positive bacteria in this study were 10 μg norfloxacin (NOR), 10 μg gentamicin (GEN), 300 μg nitrofurantoín (NIT), 10/10 μg ampicillin/sulbactam (AMS), 1.25/23.75 μg trimethoprim/sulfamethoxazole (SXT), 30 μg nalidixic acid (NA), 5 μg ciprofloxacin (CIP),10 μg ampicillin (AMN), 30 μg amikacin (AKN), 30 μg ceftazidime (CAZ), 30 μg cefuroxime (CXM), and 10 μg imipenem (IMP). In addition, ATB specifically targeting Gram-positive cocci were also tested: 1 μg oxacillin (OXA), 15 μg erythromycin (ERY), clindamycin (CLI) 2 μg, 30 μg vancomycin (VAN), and 30 μg cefoxitin (FOX). Inhibition halos were measured and interpreted according to the Clinical and Laboratory Standards Institute (CLSI) guidelines (CLSI, 2020). Multi-drug resistance (MDR) was defined as acquired non-susceptibility to at least one agent in three or more antimicrobial categories (Siegel et al., 2006).

### Detection of genes encoding virulence factors

The presence or absence of virulence-associated genes was screened using PCR. For each bacterial genus, the following genes were tested: for *E. coli*, hemolysin (*hlyA*), cytotoxic necrotizing factor (*cnf1*), fimbria type I regulator (*fimB*), type 1 fimbria (*fimA*), fimbria P (*papA*) and iron uptake related genes (*iroN* and *iutA*); for *K. pneumoniae*, hemolysin (*hlyA*), type 1 fimbrial subunit (*fimA*), the type 3 fimbrial adhesin (*mrkD*), enterobactin (*entB*), and an outer membrane porin (*ompK36*); for *E. faecalis*, gelatinase (*gelE*), sortase-type enzyme (*srt*), bacterial adherence protein (*efaA*), collagen adhesin protein (*ace*), cytolicin production activator (*cylA*); for *Staphylococcus* spp., staphylococcal enterotoxin A (*sea*), slime production related protein (*ica*), arginine metabolism related protein (*argB, argC*), lipase (*gehC*), fibrinogen binding protein (*sdrG*) and for *Bacillus* spp., bacillus enterotoxins (*entB, entA*), pore-forming toxin (*cytK*), hemolysin (*hlyIII*), sporulation related -ATPase subunit (*clpC*), capsule synthesis protein (*capA*). PCR reactions were performed using the primers and parameters described in Supplementary Table S1. The PCR reaction mixture (25 μL total) consisted of 12.5 μL of 2 × SYBR Green PCR Master Mix (Bio-Rad), 300 nmol of each forward and reverse primer, and 10 ng of genomic DNA as the template. The amplification protocol consisted of an initial denaturation step at 95°C for 4 min, followed by 35 cycles of denaturation at 95°C for 15 s and annealing/extension at 56°C for 30 s. Fluorescence signals were recorded at the end of each extension step, and a melt curve analysis was performed to verify the specificity of the product. To confirm amplification, 5 μL of the PCR product was analyzed on a 2% agarose gel, with a 100 bp DNA ladder (Promega) included for size reference. Positive and negative controls were included, consisting of a 16S gene and a free-template tube, respectively.

### Bacterial interaction assays: colony interaction, liquid medium co-culture, and mixed biofilm formation

Bacterial interaction was evaluated in co-isolated pairs obtained from the same catheter. Four co-isolated pairs were selected, consisting of *Bacillus* strains with other UP: *E. faecalis* Ef5/*B. pumilus* Bp1, *S. epidermidis* Se3/*B. subtilis* Bs1, *S. epidermidis* Se4/*B. subtilis* Bs2, and *K. pneumoniae* Kp2/*B. megaterium* Bm2. For colony interaction assays, a working protocol developed in the laboratory was followed. Briefly, bacteria were grown overnight (ON) at 37°C in a BHI liquid medium with shaking (150 rpm). Then, 5 μL aliquots of the cultures were taken and placed on the surface of a solid BHI-agar medium plate, following the scheme of one central bacterium and four concentric bacteria at different distances. Bacteria (central or peripheral) were seeded simultaneously or in a deferred manner (24 h after the growth of the central colony). The interaction was analyzed qualitatively over a period of 2 days at 30°C, and changes in colony aspects, such as growth, size, and morphology, were documented. For liquid media co-culture assays, a previously described protocol was carried out (Juarez and Galvan, 2018; Learman et al., 2019; Gaston et al., 2020). Briefly, isolates were grown ON at 37°C in BHI, harvested by centrifugation, and adequately diluted in BHI medium to an A_600 nm_ of 0.1. Monospecies or mixed cultures were grown under shaking conditions at 37°C for varying times, and viability was measured using appropriate media and ATB to facilitate single-species identification in the interaction. For mono- or multispecies biofilm assays, isolates were grown at 37°C in BHI and diluted to an A_600 nm_ of 0.1. Cells of different bacteria (alone or in co-culture) were grown in multiwell plates during 24 h at 30°C. After incubation under static conditions, the non-adherent fraction (planktonic cells) was removed, and total biofilm biomass was quantified using the crystal violet technique (O'Toole and Kolter, 1998). The non-adherent fraction containing a mixed culture was quantified by CFU mL^−1^ using the appropriate media, as mentioned earlier. To quantify microorganisms in the biofilm biomass, adhered cells were washed three times with sterile PS and incubated with 0.1% Triton X-100 for 10 min. Biofilm cells were extracted by scraping vigorously with a sterile tip. These bacteria were resuspended in sterile PS and quantified by determining CFU mL^−1^ using appropriate media, as mentioned before (Juarez and Galvan, 2018).

### Statistical analysis

Statistical analyses were performed using R (version 4.4.1) and RStudio (version 2024.09.0+375). A significance level of α = 0.05 was set for all inferential tests. Prior to applying parametric tests, the assumptions of normality and homogeneity of variances (homoscedasticity) were assessed using the Shapiro–Wilk test and Levene's test, respectively. When these assumptions were violated, such as with microbial counts spanning several orders of magnitude and exhibiting heteroscedasticity, a logarithmic transformation [log(*x*+1)] was applied to stabilize variances and improve normality. A two-way analysis of variance (ANOVA) was conducted to evaluate the effects of the independent variables and their interaction with the response variable. Model residuals were examined to verify the assumptions of normality and homoscedasticity required for the analysis of variance (ANOVA). When significant effects were detected, Fisher's Least Significant Difference (LSD) *post-hoc* test was used for pairwise group comparisons. To explore multivariate patterns and identify potential groupings among bacterial genera, a principal component analysis (PCA) was performed. The phenotypic traits of the bacterial genera were included as variables. All variables were standardized, and PCA was conducted using a correlation matrix to account for differences in measurement scales. A biplot based on the first two principal components was generated to visualize the distribution of observations and the contribution of variables to the principal components, represented by the PCA loadings. Clusters were visually identified and characterized using ellipses, and the within-cluster sum of squares (WSS) was calculated to assess the compactness of each cluster.

## Results and discussion

### Clinical isolates from double-J catheters reveal polymicrobial colonization

A total of 27 bacteria were isolated from 8 catheters (Table 1). Monobacterial isolates were obtained from three catheters (catheters 1, 4, and 5), whereas multiple bacterial species were recovered from the remaining catheters, confirming the polymicrobial etiology of the double-J stents (Chatterjee et al., 2014; Klis et al., 2014; Wang et al., 2021). The predominant bacterial species were *Bacillus* spp., including *B. pumilus, B. subtilis*, and *B. megaterium* (7 isolates, 27%), followed by *Staphylococcus* spp., including *S. aureus* and *S. epidermidis* (1 and 6 isolates, respectively, 27%). *E. faecalis* (5 isolates, 19%), *K. pneumoniae* (4 isolates, 15%), and *E. coli* (3 isolates, 12%) were also identified (Table 1). All patients had their catheters in place for a period of 1–3 months and received ATB therapy prior to extraction. Despite negative urine cultures (UC), both adhered and non-adhered bacteria were isolated from all devices. This finding aligns with several reports indicating that colonization of double-J catheters does not correlate linearly with urine culture (UC) or prostatic secretion samples (Klis et al., 2009). The identified species (except for *Bacillus* spp.) are in agreement with prior literature concerning bacterial species in catheters, which describes them as common urinary tract infection (UTI) pathogens (Gould et al., 2010; Kart et al., 2017; Pérez et al., 2017; Al-Qahtani et al., 2019). Although only 12% of the isolates were UPEC, this bacterium is the most common pathogen in both community-acquired and nosocomial urinary tract infections, accounting for approximately 24%−39% of catheter-associated urinary tract infections (CAUTIs; Jacobsen et al., 2008). It is widely known that *K. pneumoniae* is an opportunistic pathogen commonly distributed in the perineum, which can easily colonize the urinary tract and medical devices (Pérez et al., 2017). *E. faecalis* establishes a symbiotic relationship with other UP and is frequently found in the context of CAUTIs (Salm et al., 2023). Additionally, *S. aureus* and *S. epidermidis*, which are normally present on the skin surface, can enter the urinary tract during catheter placement (Klis et al., 2014; Badhan et al., 2016; Oliveira et al., 2018). Often, the cause of this colonization may be that catheterization and its manipulation promote ascending infections due to the presence of commensal microorganisms in the surrounding skin of the urethra or potential contamination resulting from the manipulation of healthcare personnel (Kalsi et al., 2003; Chatterjee et al., 2014). Notably, several bacterial isolates adhered to different catheters belonging to the *Bacillus* genus. It is well-known that human infections caused by *Bacillus* spp., except for *B. anthracis* or *B. cereus*, are rarely reported in the literature. The primary reason for this can be attributed to the fact that most of these bacteria are often considered laboratory contaminants with little to no clinical relevance (Drobniewski, 1993; Fekete, 2015). However, some reports described that *Bacillus* spp. has been isolated from bacteremia, endocarditis, wounds, respiratory, urinary, and gastrointestinal tract infections, food poisoning, and meningitis (Drobniewski, 1993; Fekete, 2015). Therefore, the identification of aerobic Gram-positive spore-forming bacilli at the species level, the evaluation of their pathogenic potential, and the interpretation of susceptibility ATB tests for these bacilli would be relevant in elucidating their potential role in the mentioned infections (Klis et al., 2014; Kandi et al., 2016).

**Table 1:** Clinical isolates obtained from double-J catheters.

**Name**	**Identification**	**Source**
Ec1	*Escherichia coli*	Catheter 2—Adhered
Ec2	*Escherichia coli*	Catheter 2—Non-adhered
Ec3	*Escherichia coli*	Catheter 2—Non-Adhered
Kp1	*Klebsiella pneumoniae*	Catheter 8—Adhered
Kp2	*Klebsiella pneumoniae*	Catheter 8—Adhered
Kp3	*Klebsiella pneumoniae*	Catheter 8—Adhered
Kp4	*Klebsiella pneumoniae*	Catheter 8—Adhered
Ef1	*Enterococcus faecalis*	Catheter 2—Adhered
Ef2	*Enterococcus faecalis*	Catheter 2—Adhered
Ef3	*Enterococcus faecalis*	Catheter 2—Adhered
Ef4	*Enterococcus faecalis*	Catheter 2—Non-adhered
Ef5	*Enterococcus faecalis*	Catheter 3—Adhered
Ef6	*Enterococcus faecalis*	Catheter 3—Adhered
Sa1	*Staphylococcus aureus*	Catheter 1—Adhered
Se1	*Staphylococcus epidermidis*	Catheter 4—Adhered
Se2	*Staphylococcus epidermidis*	Catheter 5—Adhered
Se3	*Staphylococcus epidermidis*	Catheter 6—Adhered
Se4	*Staphylococcus epidermidis*	Catheter 7—Adhered
Se5	*Staphylococcus epidermidis*	Catheter 6—Non-adhered
Se6	*Staphylococcus epidermidis*	Catheter 7—Non-adhered
Bm1	*Bacillus megaterium*	Catheter 4—Non-adhered
Bm2	*Bacillus megaterium*	Catheter 8—Adhered
Bp1	*Bacillus pumilus*	Catheter 3—Adhered
Bs1	*Bacillus subtilis*	Catheter 6—Adhered
Bs2	*Bacillus subtilis*	Catheter 7—Adhered
Bs3	*Bacillus subtilis*	Catheter 7—Non-adhered
Bs4	*Bacillus subtilis*	Catheter 6—Non-adhered

Our data showed a high percentage of bacterial catheter colonization. Several authors have reported that the catheter colonization rate exceeds the urinary infection rate, indicating a significant inconsistency between urinary infections and catheter colonization. This discrepancy complicates the estimation of stent colonization (Kehinde et al., 2004; Klis et al., 2009). Therefore, a thorough study should be conducted regarding the prevalence of etiological agents and their antimicrobial susceptibility in double-J catheters to improve treatments for long-term catheterized patients.

### Clinical isolates exhibit multiple virulence-associated phenotypes that support potential catheter colonization

Measuring experimental phenotypic outcomes could predict the UP potential infectivity. To understand the virulence strategies of the isolated strains, we performed an extensive set of phenotypic microbiological assays, which included evaluating biofilm formation capacity, amyloid-type fiber production, cellulose, and mucoid substance production as components of the extracellular matrix, motility, siderophore production, and hemolytic capacity, along with their ATB resistance profile. These phenotypes are summarized in Table 2 and described below.

**Table 2:** Virulence associated-phenotypes.

**UP isolate**		**Biofilm formation**						**Amyloid-type fibers production**	**Cellulose production**	**Motility**	**Hemolytic capacity**	**Siderophore production**	**MDR phenotype**
		**Culture conditions**											
		**M63**	**LB**	**MacConkey**	**TSB**	**BHI**	**Urine**	**LB**+**CR**+**BB**	**LB**+**CW**	**0.3%BHI**	**Blood agar**	**CAS**	**MH**
*E. coli*	Ec1	**+**	**+**	**-**	**++**	**-**	**++**	**+**	**-**	**+**	γ	**+**	**+**
	Ec2	**++**	**–**	**–**	**+++**	**+**	**++**	**+**	**–**	**+**	α	**+**	**+**
	Ec3	**+++**	**–**	**–**	**++**	**+**	**+++**	**–**	**–**	**+**	γ	**+**	**+**
*K. pneumoniae*	Kp1	**+++**	**++**	**+++**	**+++**	**++**	**+++**	**–**	**+**	**–**	γ	**–**	**+**
	Kp2	**++++**	**+**	**++++**	**++++**	**+**	**++++**	**–**	**+**	**–**	γ	**+**	**+**
	Kp3	**++++**	**++**	**+**	**+++**	**++**	**++++**	**–**	**+**	**–**	γ	**–**	**+**
	Kp4	**++++**	**+**	**++++**	**++++**	**+**	**++++**	**–**	**+**	**–**	γ	**+**	**+**
*S. aureus*	Sa1	**+**	**+**	**+++**	**+**	**++**	**+++**	**+**	**+**	**+**	α	**–**	**–**
*S. epidermidis*	Se1	**–**	**+**	**–**	**+**	**+**	**+++**	**+**	**+**	**++**	α	**–**	**+**
	Se2	**–**	**+**	**–**	**+++**	**+++**	**+++**	**+**	**+**	**++**	α	**–**	**+**
	Se3	**–**	**–**	**–**	**–**	**++**	**+++**	**+**	**+**	**++**	γ	**–**	**+**
	Se4	**–**	**–**	**+++**	**+++**	**++**	**+++**	**+**	**+**	**–**	α	**+**	**–**
	Se5	**++**	**+**	**++++**	**++**	**+**	**+++**	**+**	**–**	**–**	β	**–**	**–**
	Se6	**–**		**–**	**++**	**+**	**++**	**+**	**+**	**++**	α	**–**	**+**
*E. faecalis*	Ef1	**+++**	**+**	**++++**	**+++**	**++**	**+++**	**–**	**+**	**+**	α	**–**	**+**
	Ef2	**+++**	**–**	**+++**	**+++**	**+++**	**+++**	**–**	**+**	**+**	α	**–**	**+**
	Ef3	**++**	**–**	**++**	**+++**	**++**	**+++**	**–**	**+**	**++**	α	**–**	**+**
	Ef4	**+++**	**–**	**+**	**+++**	**+++**	**+++**	**–**	**+**	**+**	α	**–**	**+**
	Ef5	**+++**	**+**	**+++**	**++**	**++**	**+++**	**–**	**+**	**+**	α	**–**	**+**
	Ef6	**+++**	**+**	**++**	**++**	**+++**	**+++**	**–**	**+**	**–**	γ	**–**	**+**
*B. pumilus*	Bp1	**–**	**+**	**–**	**++**	**++**	**+++**	**+**	**+**	**+**	β	**–**	**+**
*B. megaterium*	Bm1	**–**	**+**	**–**	**++**	**++**	**+++**	**+**	**+**	**–**	γ	**–**	**–**
	Bm2	**++**	**+**	**–**	**++**	**++**	**+++**	**–**	**–**	**–**	γ	**+**	**–**
*B. subtilis*	Bs1	**++**	**++**	**–**	**++**	**+++**	**+++**	**+**	**+**	**+**	β	**+**	**–**
	Bs2	**++**	**++**	**–**	**–**	**+++**	**+++**	**+**	**+**	**+**	β	**+**	**–**
	Bs3	**+**	**++**	**–**	**–**	**+++**	**+++**	**+**	**+**	**+**	β	**+**	**–**
	Bs4	**+**	**++**	**–**	**–**	**+++**	**+++**	**+**	**+**	**+**	α	**+**	**–**

Phenotypes were represented as follows:

**Biofilm formation:** –, non-biofilm producers; **+**, weak biofilm producers; ++, moderate biofilm producers; +++, strong biofilm producers; and ++++, robust biofilm producers.

**Amyloid-like fiber:** +, presence; –, absence.

**Cellulose production:** +, presence; –, absence.

**Siderophore production:** +, presence; –, absence.

**Motility: –**, no motile; +, colony diameter between 0.5 and 1 cm; and ++, colony diameter more than 1 cm.

**Hemolytic capacity:** α, partial lysis; β, complete lysis; γ, no hemolysis.

**MDR phenotype:** +, MDR; –, No MDR.

#### Biofilm formation

Since biofilm development is influenced by environmental factors, such as nutrient availability, surface type, and shear forces, assessing this trait in multiple growth media will allow us to infer the versatility of bacterial populations and their potential to form biofilms in various clinical contexts. Biofilm formation was tested in M63, MacConkey, BHI, TSB, and urine culture media at various time points. Results displayed in Table 2 show that the biofilm formation ability of *E. coli, S. epidermidis*, and *Bacillus* spp. strains varied depending on the culture medium used, being higher in TSB, BHI, and urine medium. *S. aureus, E. faecalis*, and the majority of the *K. peumoniae* isolates were considered strong or robust biofilm formers in all tested media. Differences in the ability to form biofilm among genera and conditions are typical in clinical isolates (Grillo-Puertas et al., 2015; Del Pozo, 2018; Uruen et al., 2020). It is well-known that biofilm formation is one of the most relevant virulence mechanisms used by UP during UTIs (Del Pozo, 2018; Klein and Hultgren, 2020; Lenchenko et al., 2020). Indeed, it has been reported that microorganisms living in these structures are even more virulent and resistant to ATB. Therefore, the ability of UP to form biofilms in urinary catheters and the urothelium is a crucial factor for the persistence and recurrence of UTI (Vuotto et al., 2017). Additionally, the release of bacteria from biofilms into the bloodstream can lead to widespread infections, especially in immunocompromised patients (Saint and Chenoweth, 2003; Flemming and Wingender, 2010; Lazar and Chifiriuc, 2010). The description of the isolates' biofilm-forming ability is insufficient to classify them as non-biofilm formers, as in other circumstances, such as certain clinical settings, they may have a greater capacity for adhesion and biofilm formation. Studying biofilm formation under different *in vitro* conditions provides valuable insights into the adaptability and persistence strategies of clinical isolates. Such variability may reflect the ability of these organisms to persist on medical devices or within host niches, even under fluctuating conditions. In addition to visualizing the biofilm structure, qualitative analysis was performed using confocal laser scanning microscopy (CLSM) and scanning electron microscopy (SEM) on four selected UP isolates in BHI medium (Supplementary Figures S1, S2). Results showed that *S. aureus* Sa1, *E. coli* Ec1, *K. pneumoniae* Kp2, and *E. faecalis* Ef5 strains were able to adhere to the glass surface. Although the surface coverage of Sa1 and Ec1 was relatively lower compared to Kp2, all these strains demonstrated a strong adhesion, presenting characteristic agglomerates. The biofilm surface of the Sa1, Ec1, and Kp2 strains showed a “Christmas tree forest” appearance with internal water channels. These structures, characterized by their towering growth and interconnected channels, allow for efficient exchange of nutrients and waste, enabling the biofilm to sustain itself over time (Quan et al., 2022). It is worth mentioning that the Ef5 isolate was not able to form a structured biofilm. Thus, it was not possible to reconstruct the 3D structure. In SEM assays (Supplementary Figure S2), the Ec1 strain showed a significant coverage of the glass surface. In addition, a delicate layer encapsulating groups of cells interspersed by channels was observed (indicated by an arrow in the insert), probably indicating the presence of an extracellular matrix, as previously described for this genus (Cui et al., 2020). In the Ef5 isolate, a large number of cell clusters surrounded by abundant extracellular material were found. Finally, the Kp2 isolate formed a strong biofilm with protrusions that rose from the surface, as observed by CLSM. Pili, curli, or nanotube-like structures were observed, with abundant extracellular matrix surrounding the bacteria (arrows in inserts of Supplementary Figure S2). These appendages have been reported to play essential roles during cell adhesion and biofilm formation (Busscher et al., 2008; Rodrigues and Elimelech, 2009). Regarding the Sa1 isolate, it was unable to form a structured biofilm in this assay, as dispersed cells were observed (Supplementary Figure S2). The discrepancies observed in the biofilm formation ability among the different isolates on polystyrene and glass surfaces were not surprising since surface topography is a parameter that significantly influences microbial adhesion (El Abed et al., 2012).

#### Colony morphotypes

The assessment of colonies' morphology and dye-binding capacity can be used to describe, identify, and characterize microorganisms, serving as an indicator of microbial physiology and metabolism. Binding to CR and BB dyes, as well as the interpretation of the obtained results, is specific to each bacterial genus. Table 2, Supplementary Figure S3 show the phenotypes of bacterial colonies obtained on LB plates supplemented with CR and Brilliant Blue. When curli or other amyloid-like fibers were studied, it was observed that only *Escherichia, Staphylococcus*, and *Bacillus* exhibited a colony morphotype characteristic of fiber or adhesion-type component production. Based on the morphotype classification, *E. coli* isolates showed a *ras* phenotype (Supplementary Figure S3), indicating moderate production of fimbriae curli and cellulose. *S. epidermidis* isolates Se1, Se2, Se3, and Se6 exhibited clear, flat, circular colonies with rough edges at 24 h, which turned red, wrinkled, and irregularly shaped by 96 h (Supplementary Figure S3). According to the colony morphotype, *Staphylococcus* spp. isolates were considered as matrix producers (Arciola et al., 2002). In contrast, *Bacillus* isolates Bp1, Bs1, Bs2, Bs3, and Bs4 exhibited dark or red colonies with central black halos and rough, irregular, and lobed margins (Supplementary Figure S3). This morphotype indicates the production of TasA proteins (Romero et al., 2010), which are the major amyloid-type proteins of the biofilm matrix in *Bacillus* spp. (Chapman et al., 2002; Branda et al., 2006; Bohning et al., 2022). *K. pneumoniae* or *E. faecalis* strains were characterized by white colonies with pink or reddish centers and a mucoid appearance (Supplementary Figure S3). The distinctive mucoid morphology of *K. pneumoniae* isolates indicated a high capsule production (Table 2). This phenotype was reported as one of the main virulence factors of *K. pneumoniae* involved in adhesion to host cells and biofilm formation (Vernet et al., 1995; Cavalcanti et al., 2019). The presence of a pigmented ring that stands out in the Ef5 isolate was consistent with biofilm formation capacity (Torres-Rodríguez et al., 2020).

Cellulose production is another relevant extracellular matrix component for biofilms and several virulence traits, as it provides an important physical barrier to antimicrobials (Matz et al., 2008; Mulcahy et al., 2008; Hansen and Vogel, 2011). In addition to the observed RC colony morphotypes, cellulose production was also visualized on LB-agar plates supplemented with CW, as described in the Materials and Methods section. As shown in Table 2, except for *E. coli*, all bacterial genera produced cellulose, as evidenced by the formation of fluorescent colonies when they were irradiated with UV light (Supplementary Figure S3). No fluorescence was observed in *E. coli* colonies after irradiation; only the Ec3 isolate showed slight fluorescence in the center of its colony, as expected for its *ras* morphotype.

Colony morphology depends on numerous environmental factors, such as media composition, temperature, and humidity, among others, which lead to the expression of specific genes or intercellular communication processes that control this process (Bokranz et al., 2005; Da Re and Ghigo, 2006; Kaiser et al., 2013; Sydow et al., 2022). Analysis of these components under various conditions could determine the potential high capacity of the isolates to form a biofilm in UTIs. Although the correlation between morphology, expression of extracellular components, and biofilm formation capacity has been noted in some bacterial isolates (i.e., *B. subtilis* and *S. epidermidis* strains), it has not been generalized across all bacterial genera. Although *K. pneumoniae* and *E. faecalis* strains exhibited a strong biofilm-forming ability, they did not produce amyloid-type fibers under the tested conditions (Table 2).

#### Bacterial motility

In some pathogenic bacteria, motility plays a crucial role in the initial phase of infection (Josenhans and Suerbaum, 2002), likely to overcome the electrostatic repulsion between cells and surfaces (Pratt and Kolter, 1998; Walker et al., 2007). In addition, motility contributes to the colonization of different environmental niches by facilitating the spread of the infectious agent, which is crucial in the context of UTI (Josenhans and Suerbaum, 2002). As observed in Table 2, all isolates exhibited significant motility capacity, except for *K. pneumoniae, S. epidermidis strains* Se4 and Se5, *B. megaterium*, and *E. faecalis* strain Ef6. Although both *S. aureus* and *S. epidermidis* lack flagella and, therefore, are considered non-motile bacteria, it was observed that Sa1, Se1, Se2, and Se3 isolates exhibited significant motility. Recent studies have reported flagellum-independent forms of motility in microorganisms of this genus (Pollitt and Diggle, 2017). For instance, spreading involves a sliding movement, where bacteria spread radially outward from an inoculation site, forming multiple layers of densely packed cells (Henrichsen, 1997; Kaito and Sekimizu, 2007). *E. faecalis* is also regarded as a non-motile bacterium due to the absence of flagella. However, in this study, most *E. faecalis* isolates showed motility, with the exception of the Ef6 strain. It demonstrated an *E. faecalis* migration mechanism, where the synthesis and secretion of extracellular polysaccharides were required (Ramos and Morales, 2019). Therefore, when cells are grown on semisolid agar, they penetrate and invade the medium, creating a “colony impression” (Ramos and Morales, 2019). This mechanism is relevant for translocation through monolayers of human epithelial cells, conferring adaptive advantages during infection, as it enables them to translocate from the urinary tract to the bloodstream and colonize distant anatomical sites (Muscholl-Silberhorn et al., 2000; Ubeda et al., 2010; van der Heijden et al., 2014). In general, it has been described that environmental isolates of *B. subtilis* exhibit robust motility (Kearns and Losick, 2003), in agreement with the exacerbated motility shown by the strains used in this study. There is a complex relationship between motility and biofilm formation, which depends on environmental conditions or bacterial requirements. A study conducted on *B. cereus* strains reported that bacterial motility influenced biofilm formation through three mechanisms: (1) motility is necessary for the bacteria to reach adequate surfaces to form biofilm; (2) motility promotes the recruitment of planktonic cells to invade the preformed biofilm; and (3) motility is involved in biofilm spreading and propagation (Houry et al., 2010). Based on the observed motility of *B. subtilis*, it could be assumed that motility is necessary for the bacteria to reach adequate surfaces to form biofilm (Dunne, 2002; Grillo-Puertas et al., 2015).

#### Siderophore production

Iron, a vital nutrient required for bacterial growth, is highly restricted within human hosts (Hood and Skaar, 2012; Subashchandrabose and Mobley, 2015). Most UP strains encode several iron acquisition systems, such as siderophores, to acquire iron sequestered by the host (Garcia et al., 2011). Here, we tested siderophore production by the studied clinical isolates using the CAS assay described previously. All *E. coli* and *B. subtilis strains*, including Kp2 and Kp4, Se4, and Bm1, were able to produce siderophores (Table 2, Supplementary Figure S4A). It was reported that siderophore production by different UP is an important characteristic that contributes to the potential virulence of these isolates, relevant in the catheter context (Danese et al., 2000; Yue et al., 2003; Zou et al., 2023).

#### Hemolytic capacity

Hemolysins are proteins secreted by bacteria that constitute another important virulence factor in UTIs. These toxins can disrupt host cell signaling cascades, altering the inflammatory response and inducing cell death (Guyer et al., 2002; Wiles et al., 2008). It is well-known that α-hemolysin, HlyA, can stimulate epithelial barrier rupture, leading to bacterial translocation from the urinary tract to the bloodstream (Whelan et al., 2023). Table 2 shows that 19.2% of the isolates exhibited total hemolysis (β hemolysis) with defined lysis zones (Supplementary Figure S4B). Partial hemolysis (α hemolysis) was carried out by 42.3% of the isolates, while the remaining 38.5% did not present hemolytic capacity. As observed in Table 2, *Staphylococcus* strains exhibited partial or total hemolysis capacity. It is well-known that hemolytic capacity is one of the most common virulence factors of coagulase-positive (*S. aureus*) and coagulase-negative (*S. epidermidis*) staphylococci (Moraveji et al., 2014). In *E. faecalis* isolates, only partial hemolysis was observed, in agreement with several studies that describe this genus as not being able to produce total hemolysis (Stepien-Pysniak et al., 2019; Torres-Rodríguez et al., 2020). It is worth mentioning that four out of the five isolates exhibiting total hemolysis belong to the *Bacillus* spp. genus. Similar results were obtained with *Bacillus* strains isolated from river water samples with fecal contamination, where all isolates presented total hemolytic capacity (Ostensvik et al., 2004). The pathogenicity of *Bacillus* spp. has been poorly investigated, except for *B. cereus*, a known pathogen whose pathogenic potential has been related to the secretion of several virulence proteins, such as hemolysins phospholipases, cytotoxin K (CytK) and proteases (Ramarao and Sanchis, 2013; Jessberger et al., 2015), and diverse motility factors, such as those involve in swimming and swarming (Senesi et al., 2010).

Taken together, the ability to form biofilms and produce extracellular components, such as capsules and cellulose, along with virulence factors like hemolysins and siderophores, are critical elements in the pathogenicity of UP associated with colonization of double-J catheters. These factors not only facilitate the adhesion and colonization of devices but also confer resistance to antimicrobial treatments, complicating the resolution of infections.

### Double-J catheter-associated isolates exhibit prevalence of MDR phenotype

Knowledge of the ATB susceptibility (AS) pattern of clinical isolates is necessary to characterize the microorganism, as it provides valuable context for understanding their clinical relevance and potential treatment challenges. Therefore, antibiograms using conventional ATB were performed for each isolate (Supplementary Table S2). Results in Table 2 show that *E. coli* and *K. pneumoniae* isolates exhibited a multidrug-resistant (MDR) phenotype. *E. coli* strains were resistant to 6 out of 12 antibiotics tested, with *K. pneumoniae* isolates susceptible only to imipenem and amikacin (Supplementary Table S2). Both species have frequently been reported to carry plasmids encoding extended-spectrum β-lactamases (ESBLs), which confer resistance to third-generation cephalosporins and other antibiotics of the β-lactam class (Paterson, 2006; Garau, 2008; Chen et al., 2013; Pendleton et al., 2013; Mazzariol et al., 2017). Although ESBL production was not specifically tested in our study, resistance to second- and third-generation cephalosporins supports this possibility. The preserved susceptibility to imipenem suggests that carbapenems may remain effective despite increasing reports of carbapenem-resistant strains (Logan and Weinstein, 2017). Regarding the *Staphylococcus* genus, only Se1, Se2, Se3, and Se6 showed an MDR phenotype (Table 2). Multi-drug-resistant *S. epidermidis* is increasingly recognized as a cause of opportunistic infections, particularly in patients with indwelling medical devices (Otto, 2009). These infections can be difficult to treat due to biofilm formation and the ability of *S. epidermidis* to acquire resistance genes through horizontal gene transfer. Our results are in agreement with those reported by Socohou et al. (2020), who found that *S. epidermidis* strains isolated from the surfaces of medical materials exhibited high susceptibility to gentamicin and ciprofloxacin. Regarding *E. faecalis* strains, all isolates exhibited a multidrug-resistant (MDR) profile (Supplementary Table S2). The observed resistance of *E. faecalis* isolates to ERY, CLI, OXA, and FOX is clinically concerning, particularly in hospital settings. Notably, two strains (Ef5 and Ef6) exhibited resistance to vancomycin (VAN) and erythromycin (ERY), indicating a vancomycin-resistant enterococci (VRE) phenotype. VRE infections are increasingly reported in catheterized patients and raise a serious therapeutic challenge due to limited treatment options (Rivera and Boucher, 2011; Arias and Murray, 2012; Dalhoff, 2012; Sievert et al., 2013). Resistance to macrolides and lincosamides is particularly problematic in patients allergic to β-lactams, as it restricts alternative therapeutic options. Moreover, the persistence and spread of such resistant strains increase the risk of hospital outbreaks that are difficult to control.

Results show that *Bacillus* spp. presented limited resistance to the tested ATB. Particularly, only the Bp1 strain exhibited resistance to several antimicrobials, including CAZ, ERY, CLI, OXA, and FOX, while the Bm1 and Bs3 strains were resistant only to AMP. Similar results were found in *B. pumilus* and *B. subtilis* by Adamski et al. (2023). Additionally, since most *Bacillus* species are not considered human pathogens but rather saprophytic skin microorganisms, antimicrobial susceptibility studies are typically not performed for this genus (Kalsi et al., 2003; Chatterjee et al., 2014). As environmental organisms, *Bacillus* are not frequently subjected to the selective pressures found in hospital settings, where the continuous use of ATB often leads to the development of resistance (Stenfors Arnesen et al., 2008). This limited exposure may contribute to the observed lower rates of resistance compared to other pathogens commonly encountered in healthcare environments.

### Genotypic analysis evidences the presence of key virulence determinants among clinical isolates

The ability of UP isolates to cause different diseases is mediated by multiple virulence factors, including the expression of genes associated with the production of adhesins, toxins, siderophores, and secretion systems, among others (Blanco et al., 2002; Johnson, 2002). These factors are involved in the colonization of specific host surfaces, evading immune defenses, or causing direct damage to cells and tissues (Johnson, 2002). A genotypic analysis of some virulence-associated genes facilitates the identification of different phenotypic traits that may contribute to the persistence and severity of infections. *E. coli* isolates harbored genes encoding iron acquisition systems (*iutA* and *iroN*) and the hemolysin-encoding gene *hlyA*. Additionally, the Pap fimbriae (*papA*) and cytotoxic necrotizing factor (*cnf-1*) genes were detected in all isolates, suggesting a highly virulent phenotype. The *iutA* and *iroN* genes are known to enhance bacterial survival by facilitating iron acquisition in iron-limited environments, such as the human body (Johnson et al., 2005). Kanamaru et al. (2003) reported the prevalence of putative uropathogenic virulence factors in 427 *E. coli* strains, finding a significant prevalence rate of the *iroN* gene in the UTI isolates. The presence of *hlyA* and *cnf-1* further suggests that these isolates have the potential to cause severe infections, as both genes are associated with tissue damage and inflammatory responses, as typically observed in UPEC (Rendon et al., 2007; Totsika et al., 2012). Soto et al. (2007) reported that the *papA* and *hlyA* genes were present in 42% and 36% of the UPEC strains analyzed, respectively. It is worth mentioning that 50% of UPEC produce α-hemolysin, and its expression is strongly associated with symptomatic UTIs (Pigrau, 2013).

All *K. pneumoniae* isolates were positive for all virulence genes tested (Table 3), indicating their potential to cause infections by adhering to host tissues and evading the immune system. *OmpK36* expression has been linked to ATB resistance (Wyres et al., 2020), consistent with the MDR profile previously described for this genus (Table 2, Supplementary Table S2). Additionally, the combination of adhesion factors and hemolysin suggests that these strains may be particularly adept at establishing infections, especially in immunocompromised patients (Paczosa and Mecsas, 2016).

**Table 3:** Presence of virulence factor coding genes.

**UP isolate**		**Genes**						
		***fimA***	***fimB***	***iutA***	***iroN***	***papA***	***cnf-1***	***hlyA***
*E. coli*	Ec1	–	+	+	+	+	+	+
	Ec2	–	+	+	+	+	+	+
	Ec3	–	+	+	+	+	+	+
		***fimA***	***entB***	***mrkD***	***ompK36***	***hlyA***		
*K. pneumoniae*	Kp1	+	+	–	+	+		
	Kp2	+	+	–	+	+		
	Kp3	+	+	–	+	+		
	Kp4	+	+	–	+	+		
		***sea***	***ica***	***agrB***	***argC***	***geH***	***sdrG***	
*S. aureus*	Sa1	–	–	–	+	+	+	
*S. epidermidis*	Se1	–	–	–	+	+	+	
	Se2	–	–	–	+	+	+	
	Se3	–	–	–	+	+	+	
	Se4	–	–	–	–	+	+	
	Se5	–	–	–	–	+	+	
	Se6	–	–	–	+	+	+	
		***gelE***	***srt***	***efaA***	***ace***	***cylA***		
*E. faecalis*	Ef1	+	–	+	+	+		
	Ef2	+	–	+	+	+		
	Ef3	+	–	+	+	+		
	Ef4	+	–	+	+	+		
	Ef5	+	–	+	+	+		
	Ef6	–	–	–	–	–		
		***entA***	***entB***	***cytK***	***clpC***	***sdrG***	***hlyA***	
*B. pumilus*	Bp1	–	–	–	–	–	–	
*B. megaterium*	Bm1	–	–	–	–	–	–	
	Bm2	–	–	–	–	–	–	
*B. subtilis*	Bs1	+	+	–	+	+	+	
	Bs2	–	–	–	–	–	–	
	Bs3	–	–	–	–	–	–	
	Bs4	+	+	–	+	+	+	

The *S. aureus* isolates exhibited virulence genes associated with the accessory gene regulator (agr) system, including *argC* and *geH*. This system is involved in the expression of toxins and surface proteins that aid in immune evasion and tissue invasion (Novick and Geisinger, 2008). The agr system is a well-documented key virulence regulator in *S. aureus*, controlling the production of toxins, such as hemolysins and proteases (Novick and Geisinger, 2008). Additionally, the *sdrG* gene, associated with fibrinogen-binding proteins that contribute to biofilm formation and immune evasion, was observed in this isolate. The presence of this gene indicates an enhanced biofilm-forming capacity, exhibiting strong resistance to immune clearance and ATB treatment (Kong et al., 2016). *S. epidermidis* isolates also contained the *agrC, geH*, and *sdrG* genes, all of which are associated with biofilm formation (Kavanaugh and Horswill, 2016). This ability enhances the survival of *S. epidermidis* on indwelling medical devices, such as catheters and prosthetic joints (Otto, 2009). The presence of these virulence factors suggests that these *S. epidermidis* isolates may be well-adapted and capable of causing persistent infections in healthcare settings.

Except for Ef6, all *E. faecalis* isolates were positive for *gelE, efaA, ace*, and *cylA* genes. The presence of these virulence factors highlights the pathogenic potential of *E. faecalis*. Gelatinase, encoded by the *gelE* gene, is a protease that degrades host tissues and promotes biofilm development by facilitating bacterial adhesion (Hancock and Perego, 2004). The Ace protein is involved in adhesion to extracellular matrix proteins and plays a crucial role in host tissue colonization and the formation of biofilms on medical devices (Singh et al., 2010). EfaA is also involved in bacterial adherence and is considered essential for biofilm formation during infection (Nallapareddy et al., 2005). The expression of these genes is consistent with the considerable biofilm formation observed in these isolates. Finally, cytolysin (*cylA*) is involved in the lysis of red and white blood cells, contributing to immune evasion and tissue destruction (Hancock and Gilmore, 2006). This result is in agreement with the partial hemolytic capacity exhibited by these isolates.

Among the analyzed *Bacillus* species, only *B. subtilis* (Bs1 and Bs4) exhibited *hlyA* and *sdrG* genes. This was unexpected since *Bacillus* spp. are generally regarded as environmental bacteria with limited clinical relevance (Turnbull, 1996). However, recent studies by Bianco et al. (2020) and Fayanju et al. (2024) identified specific virulence genes in *B. subtilis* strains that contribute to their pathogenicity. In addition to virulence factors, the antimicrobial resistance profiles of *Bacillus* strains play a critical role in determining their pathogenicity and potential impact on indwelling device contexts.

### Clinical isolates display differential survival under host-mimicking stress conditions

In the urinary tract, bacteria are exposed to various host-mediated stress responses, including osmotic stress, pH changes, reactive oxygen species (ROS) generated by the immune system, and nutrient limitation (Agace et al., 1995). Therefore, pathogens develop adaptive advantages to cope with these environmental stressors.

**Figure 1:**
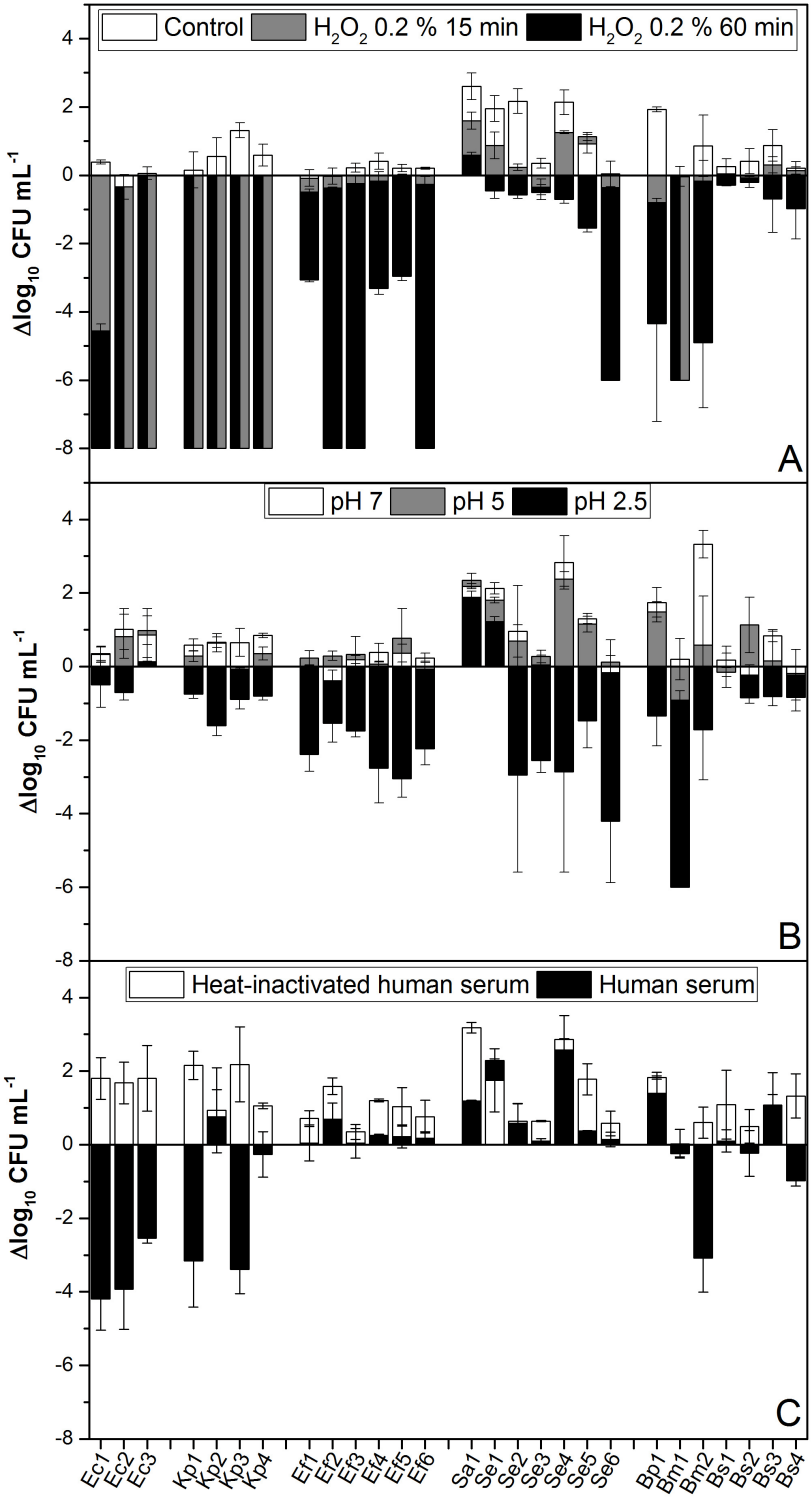
pH, H_2_O_2_, and human serum tolerance assays. **(A)** A total of 10^8^ CFU mL^−1^ of each isolate was inoculated into BHI containing 0.2% H_2_O_2_ and maintained statically at room temperature. Samples were taken and serially diluted at 0, 15, and 60 min. **(B)** From an overnight culture, the isolates were diluted to 10^8^ CFU mL^−1^ in BHI buffered to pH 7 (control), 5, or 2.5, as indicated. Cultures were incubated for 2 h at 37°C with aeration. **(C)** Isolates were cultured overnight in BHI, washed, and then resuspended in PBS. Then, 10^8^ CFU mL^−1^ was added to 100% complete human serum and heat-inactivated human serum. Samples were incubated statically at 37°C for 60 min. After incubation, the CFU mL^−1^ was determined. Data represent the mean ± SD of at least four independent experiments. A one-way ANOVA was performed with the Fisher test, yielding a *p*-value of 0.05.

All isolates were exposed to pH levels of 7, 5, and 2.5 for 2 h to simulate the expected pH ranges that bacteria may encounter in the gut, urine, or during neutrophil attack, respectively (Figure 1). The results showed that both Gram-positive and Gram-negative isolates exhibited significant decreases in viability when exposed to pH 2.5. Only Ec2, Sa1, Bs1, and Bs4 strains were tolerant to all tested pH conditions (Figure 1A). The decreased viability when exposed to pH 2.5 is consistent with previous findings that show that acidic conditions can severely compromise bacterial membrane integrity and metabolic function in both bacterial genera (Cotter and Hill, 2003; Foster, 2004).

To study oxidative stress, the survival of the tested isolates exposed to H_2_O_2_ was evaluated. *E. coli* and *K. pneumoniae* strains (both Gram-negative bacteria) were highly sensitive to 0.2% H_2_O_2_ even after 15 min (Figure 1B). However, Gram-positive bacteria were more tolerant to the mentioned stress, displaying variable susceptibility when exposed for up to 1 h (Figure 1B). The higher resistance in Gram-positive isolates could be due to differences in their cell wall structures and antioxidant defense systems (Imlay, 2013).

Complement-mediated killing is also a crucial innate immune defense that can be assessed by measuring bacterial survival in human serum. Only *E. coli* isolates Kp1, Kp3, and Se5 strains were partially susceptible to serum (Figure 1C). This is in agreement with the fact that, although the immune pressure of serum complement, all virulent pathogens capable of inducing active infections have evolved immune evasive strategies that primarily target the complement system (Sharma et al., 2020).

The significant resistance to exogenous stressors observed in many isolates implies that these bacteria may have a high survival rate during infections, potentially leading to chronic or recurrent infection.

### Principal component analysis of phenotypic diversity

**Figure 2:**
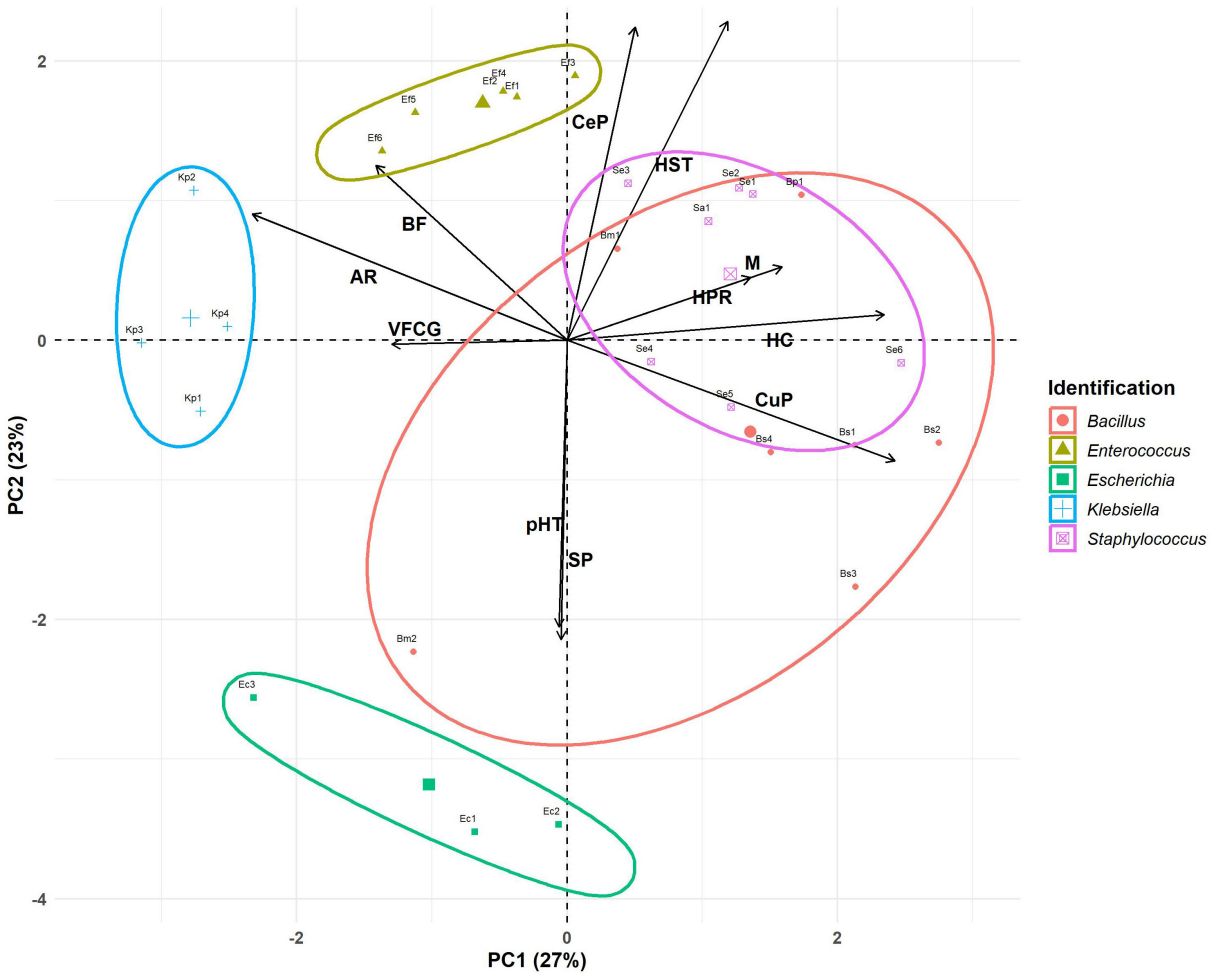
Principal components analysis. Antimicrobial resistance (AR), biofilm formation (BF), hemolytic capacity (HC), motility (M), siderophore production (SP), virulence factor coding genes (VFCG), curli production (CuP), cellulose production (CeP), H_2_O_2_ resistance (HPR), pH 2.5 resistance (pHR), and human serum resistance (HSR).

The Principal Components Analysis (PCA) plot provides a visual summary of the relationships between five different bacterial genera (color- and shape-coded by taxonomic group) based on eleven phenotypic traits: antimicrobial resistance, biofilm formation, hemolytic capacity, motility, siderophore production, virulence factor coding genes, curli production, cellulose production and H_2_O_2_, pH 2.5, and human serum tolerances (Figure 2). The variable “pH 5 resistance” was excluded from the PCA since all bacteria were tolerant (constant value). The *X*-axis (PC1) explained 27% of the variance, while the *Y*-axis (PC2) explained 23%, together accounting for 50% of the total variance, which allowed an adequate interpretation of the characteristics associated with virulence (Figure 2).

Based on PCA loadings, PC1 primarily distinguished bacteria by traits such as antimicrobial resistance (−0.45), biofilm formation (−0.27), and virulence factor coding genes (−0.25) on the left side vs. curli production (0.47), hemolytic capacity (0.46), motility (0.31), and H_2_O_2_ resistance (0.26) on the right side. PC2 differentiates bacteria based on human serum resistance (0.48), cellulose production (0.47), and biofilm formation (0.27; upper quadrant) vs. siderophore production (−0.45) and pH 2.5 resistance (−0.43; lower quadrant). It is important to highlight that biofilm formation contributes equally to both components of the PCA. Both components may reflect a balance between pathogenic potential and the host's defenses.

Each bacterial genus was enclosed within shaded ellipses, representing the clustering tendency for each bacterial group. The within-cluster sum of squares (WSS) for each group was calculated to reflect the internal variability of the clusters formed by the algorithm.

PC1 segregated the bacteria along the x-axis, positioning the clusters of *Klebsiella* (WSS: 1.53), *Enterococcus* (WSS: 1.56), and *Escherichia* (WSS: 3.12) to the left. These clusters were associated with antimicrobial resistance and the presence of virulence factor-coding genes. The distinction among these genera was marked by PC2, which separated the bacteria along the y-axis. *Enterococcus* and *Klebsiella* were located at the top of the graph, linked to biofilm formation and cellulose production. In contrast, *Escherichia* was positioned at the bottom of the graph associated with siderophore production and resistance to pH 2.5. Our results confirmed that *Klebsiella* and *Enterococcus* isolates, with robust and strong biofilm-forming abilities, respectively, were resistant to multiple antimicrobials, suggesting a direct relationship between antimicrobial resistance and biofilm-formation capacity (see Tables 1, 3). Although the UP isolates were derived from patients who had not received antibiotic therapy in the 10 days preceding the device removal procedure, it is essential to consider the potential impact of previous extended antimicrobial treatments, which may have contributed to the observed resistance. Antibiotic therapy is crucial for the treatment of UTI, but the rising prevalence of MDR UP in recent years represented a significant challenge to their effective management (Peng et al., 2018). On the other hand, the combination of increased motility with a high prevalence of virulence factors observed in *Enterococcus* strains suggested that these isolates may be particularly difficult to treat, becoming a significant risk in clinical settings.

*Staphylococcus* (WSS: 2.84) was clustered in the right center of the graph. According to PC1, this genus is associated with hemolytic capacity, motility, and curli production. Regarding PC2, this cluster was associated with cellulose production, resistance to human serum, and biofilm formation.

The most heterogeneous group was *Bacillus* (WSS: 3.99), with its members distributed across three quadrants of the biplot. Using PC1 as a reference, all members were associated with curli production (except Bm2). Regarding PC2, all members clustered based on cellulose production (except Bm2) and human serum resistance (except Bm2 and Bs3). *B. subtilis* was linked in the bottom right, associated with siderophore production and pH 2.5 resistance (PC2). Additionally, they were associated with peroxide resistance, motility, and total hemolytic capacity. Due to the *variability* of the Bacillus cluster, a key characteristic that cannot be fully appreciated is the strong biofilm formation, as all bacteria of this genus exhibit this trait. This ability could also allow it to persist in medical or industrial environments, though it is rarely associated with human infections. The high curli production and strong biofilm formation observed in both *Staphylococcus* and *Bacillus* suggest a robust capacity for adhesion and colonization on both abiotic and biotic surfaces. These microbial attributes may have implications for their virulence, potentially increasing the risk of systemic infections.

### *Bacillus* spp. modulate uropathogen behavior in polymicrobial interactions

**Figure 3:**
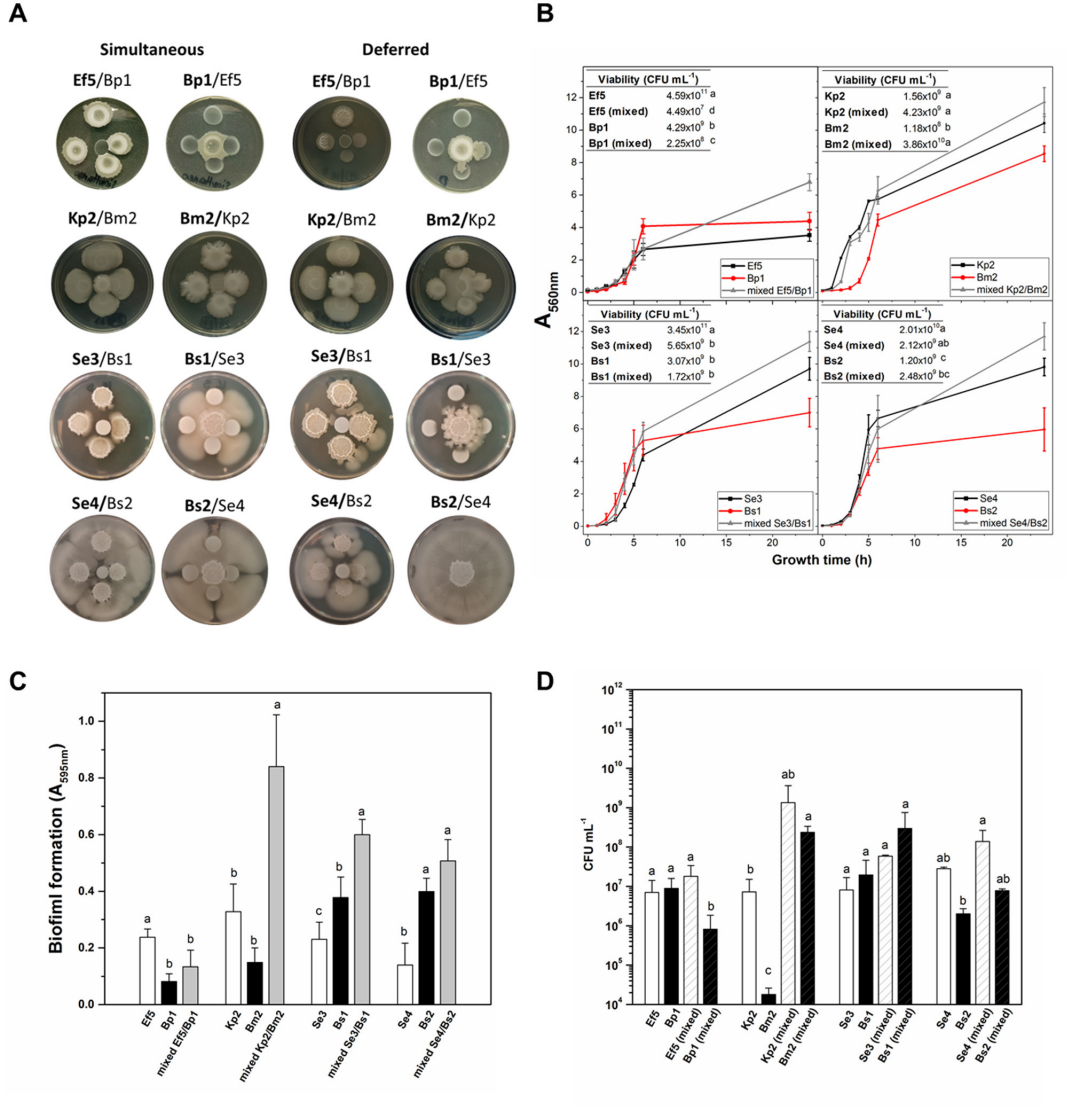
Interaction of co-isolated pairs. **(A)** In the colony interaction assay, aliquots of the co-isolated pair were inoculated simultaneously or 24 h post-growth at 30°C (deferred) on BHI-agar plates. Bold letters in the name pair indicate the central bacterium. **(B)** Growth curves (A_560nm_) of axenic or mixed (Ef5/Bp1, Kp2/Bm2, Se3/Bs1, and Se4/Bs2) were conducted in BHI with agitation for 24 h. Inset: Isolate viability in axenic or mixed conditions. **(C)** Biofilm formation (A_595nm_) and **(D)** cell viability (CFU mL^−1^) were determined for the four co-isolated pairs after 24 h of growth. Data represent the mean ± SD of at least four independent experiments. For each co-pair interaction assay, different letters indicate significant differences, as determined by one-way ANOVA performed with the Fisher test, with a *p*-value of 0.05.

Polymicrobial interactions can modify the pathogenic potential of one organism over others. Therefore, microbes co-existing in complex communities must employ diverse mechanisms, such as cross-feeding, cooperation, competition, and immune modulation, to not only shape the composition of the bacterial community but also influence interactions with the host, affecting the progression from colonization to infection (Gaston et al., 2021). Although an exhaustive characterization of each isolate grown as a pure culture was conducted, it is essential to study their behavior in polymicrobial systems. As mentioned before, in five of the eight catheters, more than one bacterial species was isolated (Table 1). Notably, *Bacillus* spp. was present in four catheters, allowing us to study its role in interactions with microorganisms typically regarded as pathogens. Therefore, we investigate the interaction between four co-isolated pairs obtained simultaneously from the same catheter: Ef5/Bp1, Kp2/Bm2, Se3/Bs1, and Se4/Bs2 (see Table 1). First, we performed interaction assays in a solid medium, with deferred and simultaneous inoculation of the isolates. As shown in Figures 3A, B, Bp1 inhibits the growth of Ef5 when inoculated simultaneously and when inoculated first in deferred assays. However, when Ef5 was first inoculated, a change in the colony complexity was observed in the interaction zone, denoted by the loss of the characteristic wrinkling of the Bp1 colony. Regarding Kp2/Bm2, Kp2 induced slight transparency in the Bm2 colony within the interaction zone, which can be interpreted as an inhibition of Bm2 during the simultaneous growth of both bacteria. This phenotype was exacerbated when Kp2 growth first occurred in the deferred assay (Figure 3A).

When *S. epidermidis*/*B. subtilis* interactions were analyzed, the results showed that, in both cases, *S. epidermidis* enhanced the motility of *B. subtilis* when the bacteria grew simultaneously. Notably, this effect was more robust in the Se4/Bs2 pair. When *B. subtilis* strains Bs1 and Bs2 were grown 24 h prior to their counterparts, they significantly inhibited the growth of Se3 and Se4, respectively (Figure 3A). The observed behaviors between these bacteria were not surprising since it was recently described that *B. subtilis* actively responds to the presence of *S. epidermidis* in its proximity by two strategies: antimicrobial production and the development of a subpopulation with a migratory response (Hernandez-Valdes et al., 2020). However, in this study, these behaviors depend on the moment in which the interaction occurs. Control of single colonies' growth at 24 and 48 h is shown in Supplementary Figure S5. Interspecies interactions vary significantly depending on whether the bacterial strains grow individually or share the same growth location. In deferred assays (e.g., Ef5, Bp1, and Bs1), the potential production of metabolites, including antimicrobial compounds or other agents that alter the local environment, may lead to the observed inhibition of the counterpart upon its arrival. However, this potential metabolite production may be modified in the presence of the other strain when both grow simultaneously, resulting in a different type of interaction. This observation raises the possibility that colonization order influences physiological adaptations and virulence expression in each species, potentially affecting the progression and severity of infection in the host.

Since bacteria in clinical settings are generally found in mixed cultures or communities that share a common environment, we decided to perform co-culture assays in agitated and static liquid media. Growth curves (A_560nm_) and viable cell counts (CFU mL^−1^) for both mixed and monocultures are shown in Figure 3B. In general, mixed cultures in all pairs, except for the Kp2/Bm2, displayed higher A_560nm_ values than monocultures after 24 h (*p* < 0.05). However, different results were observed when viable cell quantification was assessed. In mixed culture, Ef5 and Bp1 exhibited a lower viability compared to their respective monocultures (see inset tables). In the Se3/Bs1 and Se4/Bs2 pairs, the viable cells of *S. epidermidis* strains in mixed culture decreased by at least one order of magnitude, suggesting that *B. subtilis* may exert an inhibitory effect on the growth of *S. epidermidis*. This inhibition may be attributed to the production of secondary metabolites by *B. subtilis*, such as surfactins or antimicrobial peptides, which have been previously reported as antimicrobial substances (Gonzalez et al., 2011; Hernandez-Valdes et al., 2020). Conversely, in the Kp2/Bm2 pair, Kp2 viability remained unaffected when grown in mixed culture after 24 h, while Bm2 CFU mL^−1^ increased in mixed cultures.

Considering that co-isolated pairs were attached to the catheters, the biofilm formation of the four co-isolated pairs was evaluated on polystyrene plates after 24 h in mixed and axenic cultures (Figure 3C). The results showed that biofilm formation was enhanced in three of the four co-isolate pairs (Kp2/Bm2, Se3/Bs1, and Se4/Bs2) compared to single-species biofilms (Figure 3C). To assess the contribution of each isolate to biofilm formation, bacterial viability was determined in the biofilms (Figure 3D). As expected, in the co-cultures, where increased biofilm formation was observed, the number of viable cells was higher than in the monocultures. Different studies demonstrated that *B. subtilis* often facilitates biofilm development by producing extracellular polymeric substances (EPS), which provide structural support and protection to cohabitating species within the biofilm matrix (Vlamakis et al., 2013). Regarding the Ef5/Bp1 pair, Ef5 viability remained similar in mixed and single-species biofilms, while the number of viable Bp1 cells was reduced in the mixed culture compared to the monoculture (Figure 3D). This may be due to the production of antimicrobial peptides since *Enterococcus* spp. is one of the most frequent producers of bacteriocins (Almeida-Santos et al., 2021). Our results showed that interactions between the different co-isolated pairs reflected a combination of competitive, inhibitory, and cooperative behaviors in biofilm formation, consistent with the description of microbial cohabitation in this lifestyle (Luo et al., 2021).

The role of *Bacillus* spp. in these dynamics, particularly its ability to enhance biofilm formation and inhibit certain pathogens, highlights the complexity of polymicrobial interactions in complex environments, such as double-J catheters. The order of colonization may not only determine which microorganism dominates the niche but also have a direct impact on the patient's clinical outcome. If a virulent bacterium colonizes first, it may alter the environment, either promoting or limiting subsequent colonization by other species, thereby influencing the overall virulence of the infection. Conversely, simultaneous colonization could lead to complex interspecies interactions with potentially unpredictable consequences for infection progression and treatment efficacy. These findings underscore the need to investigate the role of polymicrobial colonization in clinical infections and its impact on host response and the efficacy of antimicrobial treatments.

## Conclusion

The characterization of phenotypic and genotypic traits of UP isolates obtained from double-J catheters enhances the understanding of the potential virulence of these bacteria in clinical settings. The observed virulence factors and interactions of *Bacillus* spp. suggest that this genus may serve as an opportunistic pathogen, highlighting its potential clinical relevance in co-infections of the urinary tract. Overall, this study provides a foundation for advancing knowledge of bacterial pathogenesis associated with indwelling devices, providing crucial insights for the effective clinical management and treatment of multi-bacterial urinary infections.

## Data Availability

The raw data supporting the conclusions of this article will be made available by the authors, without undue reservation.
