# Correction: Virulence traits and bacterial interactions within the complex microbial population in urinary double-J catheters

**DOI:** 10.3389/fmicb.2025.1674834

**Published:** 2025-08-13

**Authors:** Juan Vicente Farizano, Emilia Castagnaro, Julián Thomas Arroyo-Egea, Juan Daniel Aparicio, Alicia Cecilia Vallejos, Elvira María Hebert, Lucila Saavedra, Viviana Andrea Rapisarda, Josefina María Villegas, Mariana Grillo-Puertas

**Affiliations:** ^1^Instituto Superior de Investigaciones Biológicas (INSIBIO), CONICET-UNT, and Instituto de Química Biológica “Dr. Bernabé Bloj”, Facultad de Bioquímica, Química y Farmacia, UNT, San Miguel de Tucumán, Argentina; ^2^Planta Piloto de Procesos Industriales Microbiológicos (PROIMI-CONICET), Tucumán, Argentina; ^3^Hospital Eva Perón, Ministerio de Salud Pública de Tucumán, Tucumán, Argentina; ^4^Centro de Referencia Para Lactobacilos (CERELA-CONICET), Tucumán, Argentina

**Keywords:** uropathogens, catheters, biofilm, virulence, bacterial interaction, antibiotic resistance

There was a mistake in Table 1 as published. The error consists of an incorrect assignment of the source of isolates Se4 and Se5 in the published Table: Se4 was incorrectly labeled as originating from catheter 6 – non-adhered, and Se5 from catheter 7—adhered, when in fact it is the opposite.

The corrected Table 1 appears below.

**Table 1:** Clinical isolates obtained from double-J catheters.

**Name**	**Identification**	**Source**
Ec1	*Escherichia coli*	Catheter 2—Adhered
Ec2	*Escherichia coli*	Catheter 2—Non-adhered
Ec3	*Escherichia coli*	Catheter 2—Non-Adhered
Kp1	*Klebsiella pneumoniae*	Catheter 8—Adhered
Kp2	*Klebsiella pneumoniae*	Catheter 8—Adhered
Kp3	*Klebsiella pneumoniae*	Catheter 8—Adhered
Kp4	*Klebsiella pneumoniae*	Catheter 8—Adhered
Ef1	*Enterococcus faecalis*	Catheter 2—Adhered
Ef2	*Enterococcus faecalis*	Catheter 2—Adhered
Ef3	*Enterococcus faecalis*	Catheter 2—Adhered
Ef4	*Enterococcus faecalis*	Catheter 2—Non-adhered
Ef5	*Enterococcus faecalis*	Catheter 3—Adhered
Ef6	*Enterococcus faecalis*	Catheter 3—Adhered
Sa1	*Staphylococcus aureus*	Catheter 1—Adhered
Se1	*Staphylococcus epidermidis*	Catheter 4—Adhered
Se2	*Staphylococcus epidermidis*	Catheter 5—Adhered
Se3	*Staphylococcus epidermidis*	Catheter 6—Adhered
Se4	*Staphylococcus epidermidis*	Catheter 7—Adhered
Se5	*Staphylococcus epidermidis*	Catheter 6—Non-adhered
Se6	*Staphylococcus epidermidis*	Catheter 7—Non-adhered
Bm1	*Bacillus megaterium*	Catheter 4—Non-adhered
Bm2	*Bacillus megaterium*	Catheter 8—Adhered
Bp1	*Bacillus pumilus*	Catheter 3—Adhered
Bs1	*Bacillus subtilis*	Catheter 6—Adhered
Bs2	*Bacillus subtilis*	Catheter 7—Adhered
Bs3	*Bacillus subtilis*	Catheter 7—Non-adhered
Bs4	*Bacillus subtilis*	Catheter 6—Non-adhered

The original version of this article has been updated.

