# Supplementary material for: Virulence traits and bacterial interactions within the complex microbial population in urinary double-J catheters
Source: Front Microbiol. 2025 Jul 10;16:1624743. doi: 10.3389/fmicb.2025.1624743 (PMC12286952; doi:10.3389/fmicb.2025.1624743)
Supplement: Supplementary file 1 [file Data_Sheet_1.docx]

**Supplementary Material**

**Table S1. Primers used in this study.**

| **Primers** | | **Sequence 5´-3´** | | **gen** | | | **Reference** | | |  |  |
| --- | --- | --- | --- | --- | --- | --- | --- | --- | --- | --- | --- |
| ***E. coli*** | |  | |  | | |  | | |  |  |
| fimA-f | | TGCGGGTAGCGCAACAA | | *fimA* | | | Grillo-Puertas y col. 2015 | | |  |  |
| fimA-r | | ACGCAGTCCCTGTTTTATCCA | | *fimA* | | | Grillo-Puertas y col. 2015 | | |  |  |
| fimB-f | | TCTCGACTTCCGGTGGTAATG | | *fimB* | | | Grillo-Puertas y col. 2015 | | |  |  |
| fimB-r | | GCGTAACATGTGCGGATGAA | | *fimB* | | | Grillo-Puertas y col. 2015 | | |  |  |
| papA-f | | TTTTTCGGGTGTCCCAAGTG | | *papA* | | | Grillo-Puertas y col. 2015 | | |  |  |
| papA-r | | TGTTGCACCGACGGTCTGT | | *papA* | | | Grillo-Puertas y col. 2015 | | |  |  |
| cnf-1-f | | CCAGGAGGGTAAACCAGTAATCA | | *cnf1* | | | Grillo-Puertas y col. 2015 | | |  |  |
| cnf-1-r | | TTCGGGCAACAATTGTCGTA | | *cnf1* | | | Grillo-Puertas y col. 2015 | | |  |  |
| hlyA-f | | GGCACGGCGATTACTAAACAG | | *hlyA* | | | Grillo-Puertas y col. 2015 | | |  |  |
| hlyA-r | | CGTTCGGTGAGGCCAATG | | *hlyA* | | | Grillo-Puertas y col. 2015 | | |  |  |
| iutA-f | | AGCCGCAGCAATCGAACT | | *iutA* | | | Grillo-Puertas y col. 2015 | | |  |  |
| iutA-r | | CATTTTCGATAACCCAGGTGGTT | | *iutA* | | | Grillo-Puertas y col. 2015 | | |  |  |
| iroN-f | | CAGTCTGAATCTTTCGCAGGAA | | *iroN* | | | Grillo-Puertas y col. 2015 | | |  |  |
| iroN-r | | GGGCAACCCCTGCTTTG | | *iroN* | | | Grillo-Puertas y col. 2015 | | |  |  |
| ***K. pneumoniae*** |  | | | | |  | | |  | | |
| hlyA-f | GTGTCTCCCATAACCTGTACC | | | | | *hlyA* | | | This work | | |
| hlyA -r | CGTTCAAAATCACCGAAGCG | | | | | *hlyA* | | | This work | | |
| fimA-f | AGGAGAAGTCGTTAATGCGG | | | | | *fimA* | | | This work | | |
| fimA-r | AGCTGGATGTTGAAACCGAC | | | | | *fimA* | | | This work | | |
| mrkD-f | CGAAAAGACCGGAAATTCTGC | | | | | *mrkD* | | | This work | | |
| mrkD-r | GAGGCAGGGTGATATAACGAG | | | | | *mrkD* | | | This work | | |
| entB-Kp-f | TGGGCCTTTGAACCTTCC | | | | | *entB* | | | This work | | |
| entB-Kp-r | GGAATACCGTTCTGTTTGCAG | | | | | *entB* | | | This work | | |
| ompK36-f | ACGGTCTGCACTATTTCTCTG | | | | | *ompK36* | | | This work | | |
| ompK36-r | TGGTCGTTGATCTGGGTTTC | | | | | *ompK36* | | | This work | | |
| ***Staphilococcus spp.*** |  | | | | |  | | |  | | |
| sea-f | AAGCAAGACGTTATTTACAGGAAAA | | | | | *sea* | | | This work | | |
| sea-r | TGAATACTGTCCTTGAGCACC | | | | | *sea* | | | This work | | |
| ica-f | TCTATGCTGGATGTTAGTGCC | | | | | *ica* | | | This work | | |
| ica-r | CTTAAAAGTACTTCATGCCCGC | | | | | *Ica* | | | This work | | |
| agrB-f | CTTAAAAGTACTTCATGCCCGC | | | | | *agrB* | | | This work | | |
| agrB-r | AATATGACATAACAATGACGAATTTGC | | | | | *agrB* | | | This work | | |
| agrC-f | TTTGTTTCTATTACTGTACTTTTGAGTTTG | | | | | *agrC* | | | This work | | |
| agrC-r | GCTTCGATTTCTTGTAGCTTACG | | | | | *agrC* | | | This work | | |
| gehC-f | ACTATGACCGTGCTGTTGAG | | | | | *gehC* | | | This work | | |
| gehC-r | CATGACCATATTTAGCTGCGTG | | | | | *gehC* | | | This work | | |
| sdrG-f | CAGTCAGAACAGATGAAGAGGG | | | | | *sdrG* | | | This work | | |
| sdrG-r | ATTGCCTTCTGAGTCTAGTGC | | | | | *sdrG* | | |  | | |
| ***E. faecalis*** | | |  | |  | | |  | | |  |
| gelE-f | | | TTTCGGGTGCATCTAATCCAG | | *gelE* | | | This work | | |  |
| gelE-r | | | GTTTCTGGTTGTCCGTGTTTAC | | *gelE* | | | This work | | |  |
| srt-f | | | CCGCAACCAATACCAAAGAAC | | *srt* | | | This work | | |  |
| srt-r | | | CATCGACCTCATTTTCAAACGG | | *srt* | | | This work | | |  |
| efaA-f | | | AGCAGGGTTAACGTTAGCTG | | *efaA* | | | This work | | |  |
| efaA-r | | | CGAGTTCGTTGTCACAATTGC | | *efaA* | | | This work | | |  |
| ace-f | | | GGAACAGGCAACAGAAACAAG | | *ace* | | | This work | | |  |
| ace-r | | | TGGTGAGACATGAACTTCTGG | | *ace* | | | This work | | |  |
| cylAf | | | CCTACATCGCTAGTTTCTCCAC | | *cylA* | | | This work | | |  |
| cylA-r | | | TGCGCTTACTTCTGGAGTTG | | *cylA* | | | This work | | |  |
| ***Bacillus spp.*** | | |  | |  | | |  | | |  |
| entB-Bs-f | | | CGTGGTCTTGATTCGGTAAGG | | *entB* | | | This work | | |  |
| entB-Bs-r | | | GCGAGTTGTGAGCAATTTCTG | | *entB* | | | This work | | |  |
| entA-f | | | CTCCCATTCCTCATCGCTAAG | | *entA* | | | This work | | |  |
| entA-r | | | TTTTCCTGCGGATGTGAGAG | | *entA* | | | This work | | |  |
| cytK-f | | | GTTTCCAACCCAGTTTGCAG | | *cytK* | | | This work | | |  |
| cytK-r | | | CGCTTATACAAAACACGCCG | | *cytK* | | | This work | | |  |
| hlyIII-f | | | CCCTGTTGGTTATCGTATGGTC | | *hlyIII* | | | This work | | |  |
| hlyIII-r | | | CCACCCCATGACTAAATACACG | | *hlyIII* | | | This work | | |  |
| clpC-f | | | CGAGCAAGTAGAGGATACGAAG | | *clpC* | | | This work | | |  |
| clpC-r | | | TGGGCGATTTTAGATACAGGC | | *clpC* | | | This work | | |  |
| capA-f | | | ACCGTCATTTTCTACAGCCTC | | *capa* | | | This work | | |  |
| capA-r | | | TGTCACTTCAAAGCGTCCTG | | *capA* | | | This work | | |  |

**Figure S1**


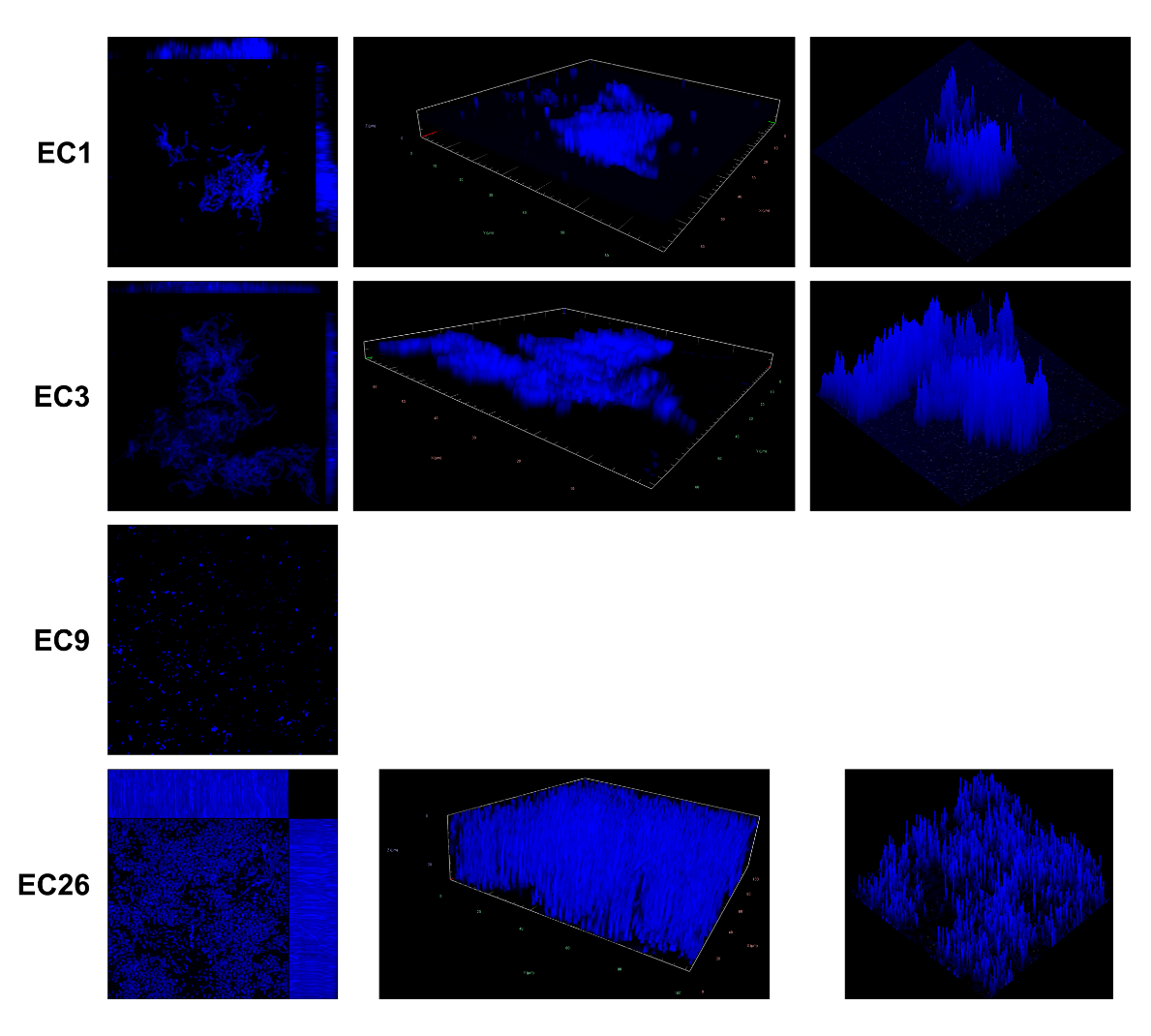


Sa1

Ec1

Ef4

Kp2

**Figure S1: Laser scanning confocal microscopy analysis.** The indicated isolates were grown in M63 medium for 72 h on specialized glass slides provided by the microscopy service. Subsequently, they were washed, and adhered cells were stained with DAPI (20 µM) for 15 minutes and fixed with paraformaldehyde. Biofilm formation was analyzed by CLSM (Zeiss LSM800), with a 63x immersion objective and the images represent three-dimensional reconstructions of the biofilm structures (ImageJ software). The images were representative of two independent experiments performed in duplicate**.**

**Figure S2**


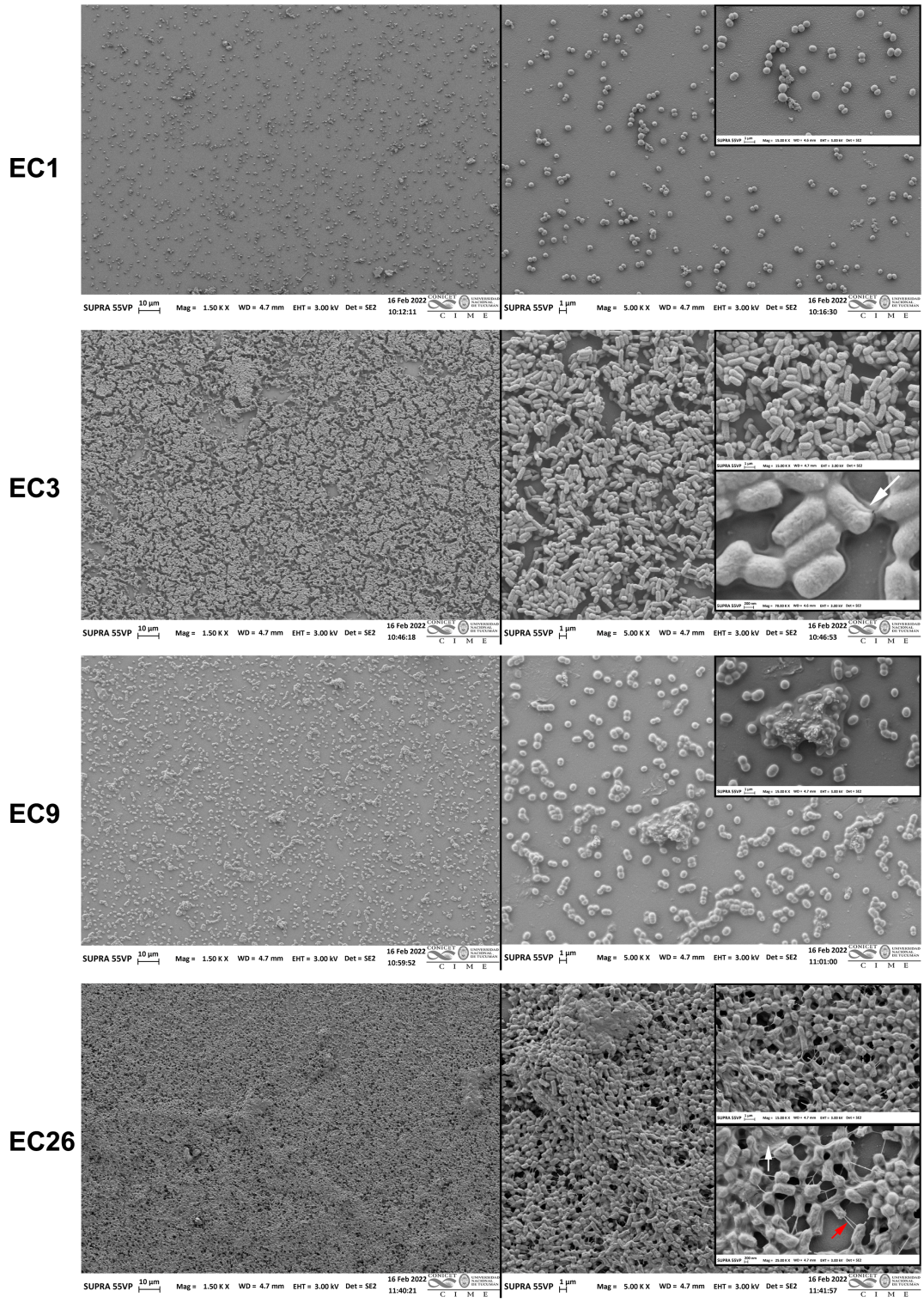


Sa1

Ec1

Ef5

Kp2

**Figure S2: Scanning electron microscopy (SEM) analysis.** The indicated isolates were grown in M63 medium for 72 h on specialized glass slides provided by the microscopy service. The slides were then washed, and adhered cells were fixed with glutaraldehyde. Biofilm formation was analyzed by SEM (Carl Zeiss SUPRA-55), with a resolution of 1.0 nm at 15 kV and 1.7 nm at 1 kV in HV (high-vacuum) mode and 2 nm at 30 kV in variable pressure (PV) mode. Magnification: Micrographs — left panels, 1500X; right panels, 5000X. Insert in EC1, 15000X; inserts in Ec1, 15000X and 70000X; insert in Ef5, 15000X; and inserts in Kp2, 15000X and 25000X. Images are representative of two independent experiments performed in duplicate.

**
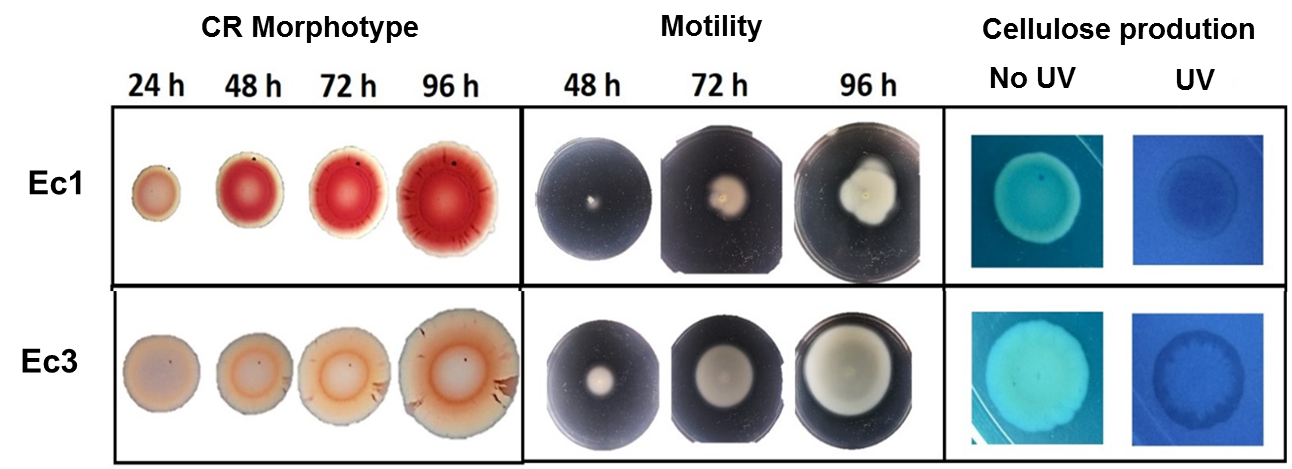
Figure S3**


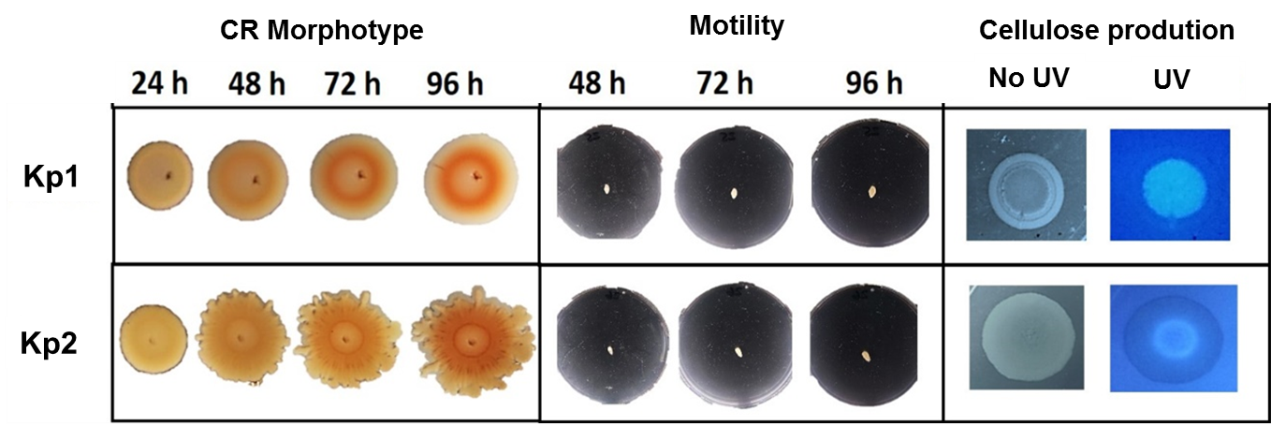


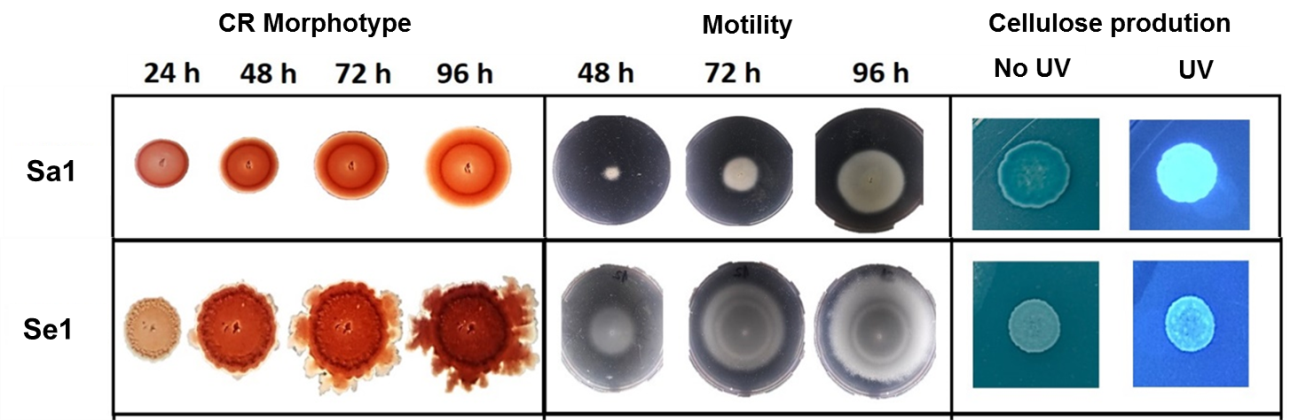


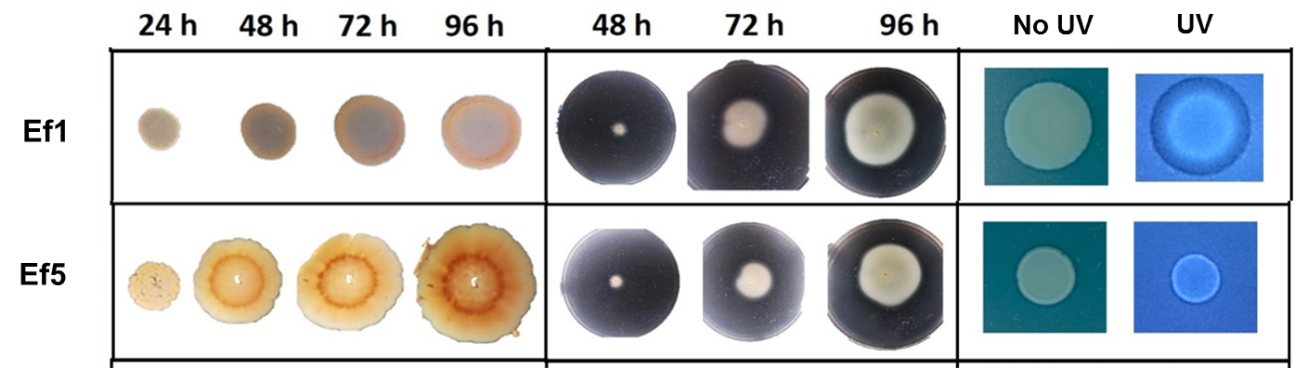


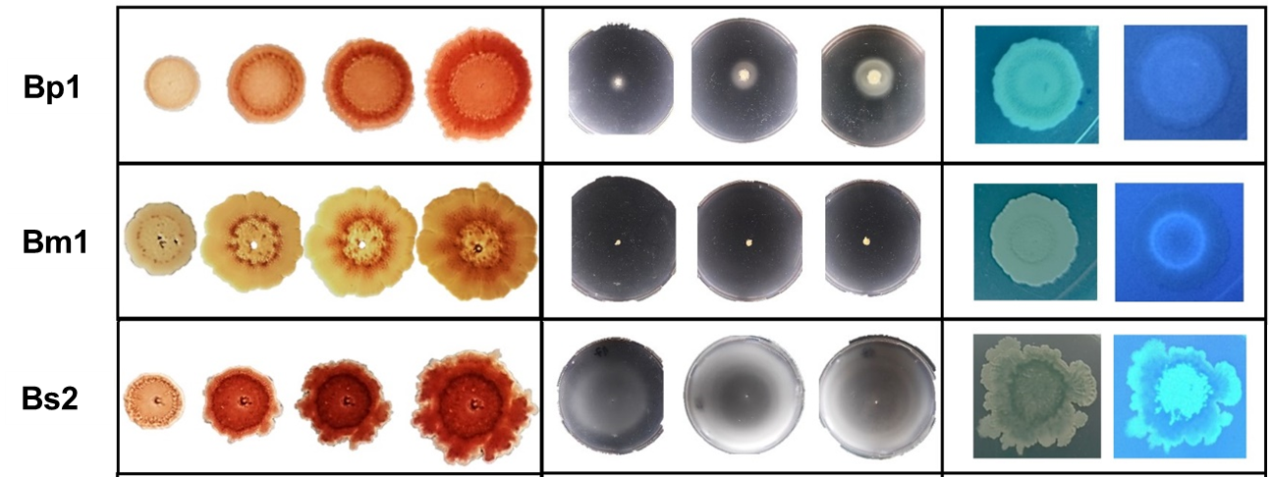


**Figure S3. Bacterial virulence-associated phenotypes**: ***curli* or amyloid fibers, cellulose, and motility**. Cultures of all isolates grown in LB or BHI medium for 24 h were washed and diluted to an A_600nm_ of 0.1. For morphology assays, suspensions were inoculated onto LB-agar plates (low Na^+^) supplemented with Congo Red (40 μg/mL) and Brilliant Blue (20 μg/mL). Motility analysis was carried out onto semi-solid M63-agar plates (0.3%) using a sterile toothpick for inoculation. Cellulose production was determined in LB-agar plates supplemented with Calcofluor White (50 μg/mL). Plates were incubated at 30°C during the indicated times for each assay and 48 h. Results are representative of four independent experiments.

**Figure S4**

**A**

**B**


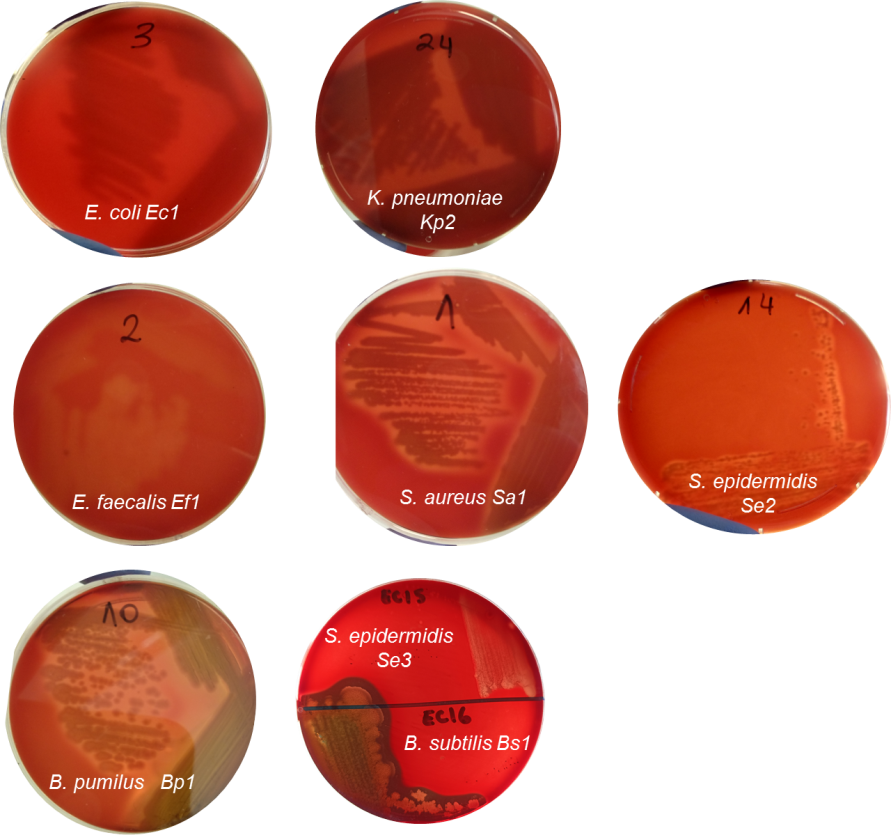

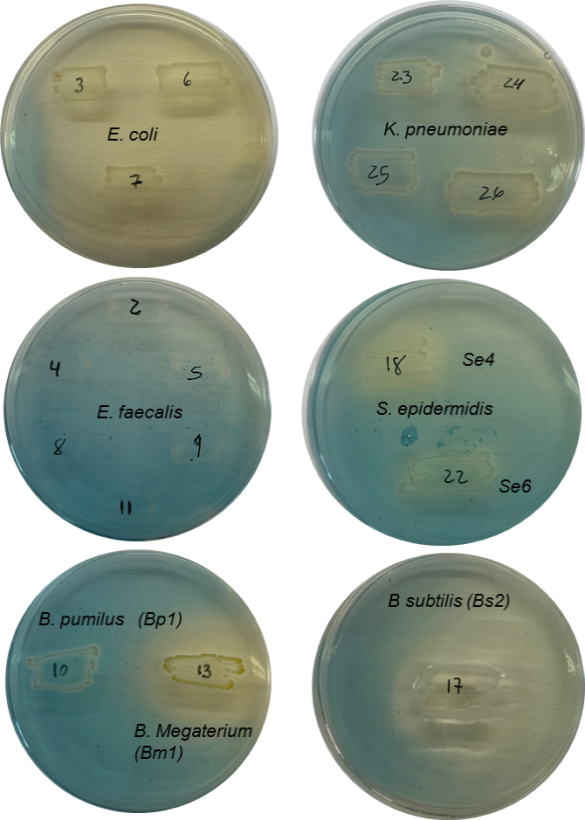


**Figure S4. Siderophores production and hemolytic activity of the isolates.** Siderophores production was evaluated by seeded isolates supentions on M9 minimal medium supplemented with 0.2% glucose plates and incubated at 30°C for 48 h. Then, an overlaid of semisolid CAS medium (see material and methods section) was poured onto the grown plate. Those isolates that are capable of producing siderophores presented a color change of the CAS medium from blue to yellowish around the colony (**A**). Hemolytic activity was evaluated on blood agar plates. Aliquots of bacterial suspensions were plated onto blood agar medium and incubated at 30°C for 24 h. Hemolysis zones were interpreted as follows: -α hemolysis: partial lysis (turbid and greenish halo), -β hemolysis: complete lysis (clear, defined halo), -γ hemolysis: no hemolysis (**B**). Results are representative of three independent experiments.

**Table S2**

| **UP isolate** |  | **Antibiotic** | | | | | | | | | | | | | | | | |
| --- | --- | --- | --- | --- | --- | --- | --- | --- | --- | --- | --- | --- | --- | --- | --- | --- | --- | --- |
|  |  | **AMS** | **SXT** | **CIP** | **FT** | **CXM** | **CAZ** | **IMI** | **GM** | **AMP** | **NA** | **NOR** | **AKN** | **ERY** | **CLI** | **VAN** | **OXA** | **FOX** |
| ***E. coli*** | **Ec1** | **R** | **R** | **R** | **S** | **S** | **S** | **S** | **S** | **R** | **R** | **R** | **S** | **-** | **-** | **-** | **-** | **-** |
|  | **Ec2** | **R** | **R** | **R** | **S** | **S** | **S** | **S** | **S** | **R** | **R** | **R** | **S** | - | - | - | - | - |
|  | **Ec3** | **R** | **R** | **R** | **S** | **S** | **S** | **S** | **R** | **R** | **R** | **R** | **S** | - | - | - | - | - |
| ***K. pneumoniae*** | **Kp1** | **R** | **R** | **R** | **R** | **R** | **R** | **S** | **S** | **R** | **R** | **R** | **S** | **-** | **-** | **-** | **-** | **-** |
|  | **Kp2** | **R** | **R** | **R** | **R** | **R** | **R** | **S** | **I** | **R** | **R** | **R** | **S** | - | - | - | - | - |
|  | **Kp3** | **R** | **R** | **R** | **I** | **R** | **R** | **S** | **I** | **R** | **R** | **R** | **S** | - | - | - | - | - |
|  | **Kp4** | **R** | **R** | **R** | **I** | **R** | **I** | **S** | **I** | **R** | **R** | **R** | **S** | - | - | - | - | - |
| ***S. aureus*** | **Sa1** | **S** | **-** | **S** | **S** | **S** | **S** | **S** | **S** | **R** | **R** | **S** | **S** | **S** | **S** | **S** | **S** | **S** |
| ***S. epidermidis*** | **Se1** | **S** | **-** | **S** | **S** | **S** | **S** | **S** | **S** | **R** | **R** | **S** | **S** | **R** | **S** | **S** | **S** | **S** |
|  | **Se2** | **S** | **-** | **S** | **S** | **S** | **S** | **S** | **R** | **R** | **R** | **S** | **S** | **R** | **S** | **S** | **S** | **S** |
|  | **Se3** | **S** | **-** | **S** | **S** | **S** | **S** | **S** | **R** | **R** | **R** | **S** | **S** | **R** | **R** | **R** | **S** | **S** |
|  | **Se4** | **S** | **-** | **R** | **S** | **S** | **S** | **S** | **S** | **I** | **R** | **R** | **S** | **S** | **S** | **S** | **S** | **S** |
|  | **Se5** | **S** | **-** | **S** | **S** | **S** | **S** | **I** | **S** | **I** | **R** | **S** | **S** | **S** | **S** | **S** | **S** | **S** |
|  | **Se6** | **S** | **-** | **S** | **S** | **S** | **S** | **S** | **S** | **R** | **R** | **S** | **S** | **R** | **S** | **S** | **S** | **S** |
| ***E. faecalis*** | **Ef1** | **I** | **-** | **R** | **S** | **R** | **R** | **S** | **R** | **S** | **R** | **R** | **S** | **R** | **R** | **S** | **R** | **R** |
|  | **Ef2** | **S** | **-** | **R** | **S** | **R** | **R** | **S** | **R** | **S** | **R** | **R** | **R** | **R** | **R** | **S** | **R** | **R** |
|  | **Ef3** | **S** | **-** | **R** | **S** | **R** | **R** | **S** | **R** | **S** | **R** | **R** | **I** | **R** | **R** | **S** | **R** | **R** |
|  | **Ef4** | **S** | **-** | **R** | **S** | **R** | **R** | **R** | **R** | **S** | **R** | **R** | **S** | **R** | **R** | **S** | **R** | **R** |
|  | **Ef5** | **S** | **-** | **R** | **S** | **R** | **R** | **R** | **R** | **S** | **R** | **R** | **R** | **R** | **R** | **S** | **R** | **R** |
|  | **Ef6** | **S** | **-** | **R** | **S** | **R** | **R** | **S** | **R** | **S** | **R** | **R** | **I** | **R** | **R** | **S** | **R** | **R** |
| ***B. pumilus*** | **Bp1** | **S** | **-** | **S** | **S** | **S** | **R** | **S** | **S** | **S** | **S** | **S** | **S** | **R** | **R** | **S** | **R** | **R** |
| ***B. megaterium*** | **Bm1** | **S** | **-** | **S** | **S** | **S** | **S** | **S** | **S** | **R** | **S** | **S** | **I** | **S** | **R** | **S** | **S** | **S** |
|  | **Bm2** | **S** | **-** | **S** | **S** | **S** | **S** | **S** | **S** | **S** | **S** | **S** | **S** | **S** | **S** | **S** | **S** | **S** |
| ***B. subtilis*** | **Bs1** | **S** | **-** | **S** | **S** | **S** | **S** | **S** | **S** | **S** | **S** | **S** | **S** | **S** | **S** | **S** | **S** | **S** |
|  | **Bs2** | **S** | **-** | **S** | **S** | **S** | **S** | **S** | **S** | **S** | **S** | **S** | **S** | **S** | **S** | **S** | **S** | **S** |
|  | **Bs3** | **S** | **-** | **S** | **S** | **S** | **S** | **S** | **S** | **R** | **S** | **S** | **S** | **S** | **S** | **S** | **S** | **S** |
|  | **Bs4** | **S** | **-** | **S** | **S** | **I** | **S** | **S** | **S** | **I** | **S** | **S** | **S** | **S** | **S** | **S** | **S** | **S** |

**Figure S5**

**
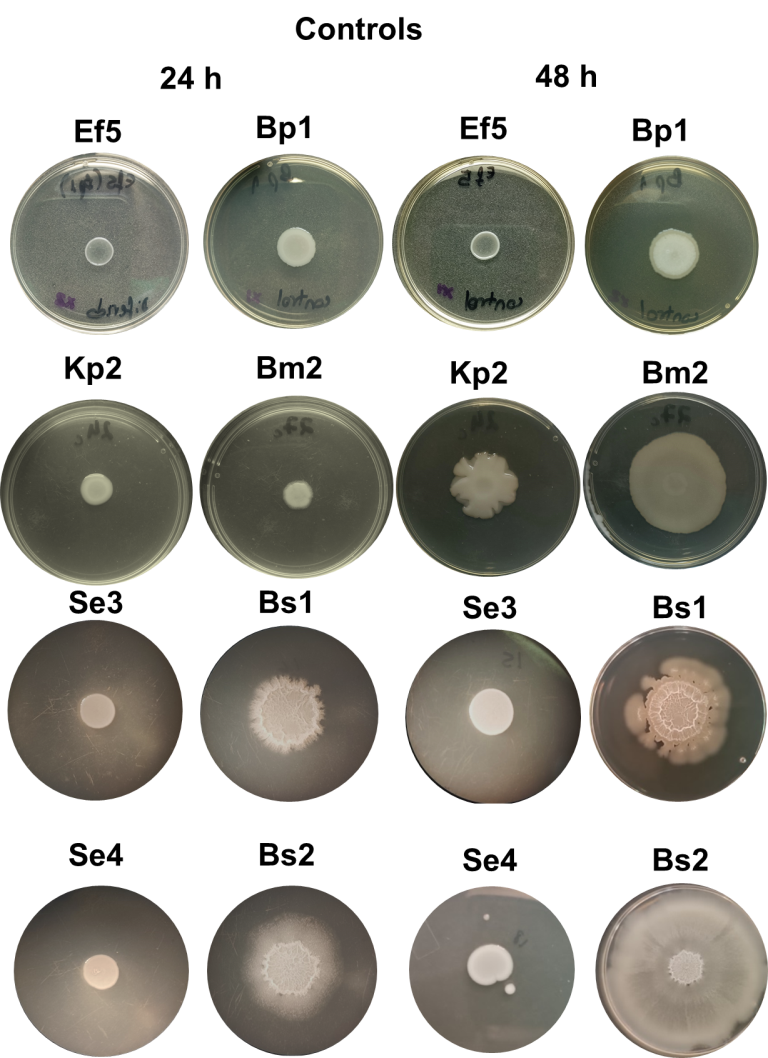
**

**Figure S5. Single colonies incubation.** 10 μl aliquots of the indicated isolates were spotted in the center of a BHI-agar plate and were grown up to the indicated times. Results represent four independent experiments.
